# Production of high protein yeast using enzymatically liquefied almond hulls

**DOI:** 10.1371/journal.pone.0293085

**Published:** 2023-11-15

**Authors:** Irnayuli R. Sitepu, Alex Hitomi, Wayne Wu, Angela Wu, Tina Jeoh, Kyria Boundy-Mills

**Affiliations:** 1 Department of Food Science and Technology, Phaff Yeast Culture Collection, University of California Davis, Davis, CA, United States of America; 2 Department of Biological and Agricultural Engineering, University of California Davis, Davis, CA, United States of America; CIFRI: Central Inland Fisheries Research Institute, INDIA

## Abstract

Animal feed ingredients, especially those abundant in high quality protein, are the most expensive component of livestock production. Sustainable alternative feedstocks may be sourced from abundant, low value agricultural byproducts. California almond production generates nearly 3 Mtons of biomass per year with about 50% in the form of hulls. Almond hulls are a low-value byproduct currently used primarily for animal feed for dairy cattle. However, the protein and essential amino acid content are low, at ~30% d.b.. The purpose of this study was to improve the protein content and quality using yeast. To achieve this, the almond hulls were liquefied to liberate soluble and structural sugars. A multi-phase screening approach was used to identify yeasts that can consume a large proportion of the sugars in almond hulls while accumulating high concentrations of amino acids essential for livestock feed. Compositional analysis showed that almond hulls are rich in polygalacturonic acid (pectin) and soluble sucrose. A pectinase-assisted process was optimized to liquefy and release soluble sugars from almond hulls. The resulting almond hull slurry containing solubilized sugars was subsequently used to grow high-protein yeasts that could consume nutrients in almond hulls while accumulating high concentrations of high-quality protein rich in essential amino acids needed for livestock feed, yielding a process that would produce 72 mg protein/g almond hull. Further work is needed to achieve conversion of galacturonic acid to yeast cell biomass.

## Introduction

Global meat supply continues to increase and is projected to reach 377 Mtons by 2031 [1,2]. Increasing demands for meat means increasing demands for high protein animal feed, a major cost contributor for livestock production [3]. Essential amino acids such as histidine, isoleucine, leucine, lysine, methionine, phenylalanine, threonine, tryptophan and valine are required to guarantee efficient livestock growth and sustainable health [3–6]. Deficiencies of these amino acids in livestock diets result in reduced growth rates, reproductive performance, and immune response [7]. Fish meal and soybean meal are two prevalent high protein feed ingredients; however, sustainable alternatives are needed to help reduce overall environmental impacts of meat production.

Plant-based products in general are associated with lower environmental impacts [8]. However, plant-based feed ingredients are low in protein and often do not fulfil stringent amino acid requirements of animal feed, and thus have a relatively low market value. For example, as of February 2023, soybean and yellow pea cattle feed are 8.2 USD/bu and 12.25 USD/bu, respectively [9,10]. In contrast, a few plant-based feedstocks provide significant quantities of protein including essential amino acids. Soybean meal is a common feed ingredient and is typically comprised of 45% crude protein, including 3% lysine, and has a market value of 432.20 USD/ton as of February 2023 [9]. Reducing the cost of producing other plant-based feed ingredients rich in protein and essential amino acids could significantly reduce the costs of livestock production. Furthermore, production of high-protein feed material with a range of amino acid composition to match the nutrition needs of various livestock at various stages of development could result in a portfolio of new sustainable feed alternatives for different types of livestock. To address this opportunity, the ability of yeasts to convert abundant agricultural residue (cassava leaves) to produce high protein material with varying concentrations of amino acids was recently demonstrated [11].

Of over 2,000 yeast species known, only three yeast species are currently used as a high protein ingredient in animal feed: brewer’s yeast (*Saccharomyces cerevisiae*), torula yeast (*Cyberlindnera jadinii*, formerly called *Candida utilis*) and *Kluyveromyces marxianus*. Different yeast species accumulate differing amounts of total protein, with differing amino acid composition [12]. Furthermore, different yeast species can utilize different carbon sources. For example, *Kluyveromyces* is produced using dairy byproducts including whey because it can consume lactose. Molasses and cane juice are popular feedstocks for industrial cultivation of *Saccharomyces cerevisiae* because this species can consume sucrose. Utilization of lower cost feedstocks would require that the yeast is able to convert the specific carbon sources in that feedstock.

In 2021, the California almond industry produced over 1.2 million US tons of almonds, plus byproducts including 2.46 million tons of almond hulls and 1.01 million tons of almond shells [13]. Almond hulls, the high carbohydrate outer layer of the almond fruits, are generally sold as a low value feed ingredient for ruminants, because of their low protein content and lack of essential amino acids necessary for cattle growth [14,15]. Like fruit hulls in general, almond hulls consist largely of soluble sucrose, insoluble structural polysaccharides such as pectin (a polymer of galacturonic acid), and varying amounts of dietary fiber, protein, and lipids [11,16,17]. Almond hulls are low value in part because they are low in protein, typically 5% weight by weight, dry basis [18]. Thus, almond hulls provide a potential low-cost input for several valorization processes, such as production of bioethanol, phenolic antioxidants, or syngas through thermal conversion [11,17,19,20].

To convert almond hulls to a high-quality animal feed supplement, improvements to the protein quantity and quality are necessary, especially essential amino acid composition. The purpose of this study was to establish an efficient bioconversion approach for production of high-quality protein animal feed from an abundant and low value waste stream from almond processing: almond hulls. Two major steps were performed to develop such a process, following a successful model from a previous study [11]. First, an enzymatic liquefaction process was developed for efficient solubilization of sucrose and other carbon sources by degradation of the pectin fraction. Second, the resulting hydrolysates were used for growing yeasts. Potential high protein yeast species from among the 1,000 yeast species preserved in the Phaff Yeast Culture Collection at the University of California Davis (https://phaffcollection.ucdavis.edu/) were screened for ability to consume the sugars found in processed almond hulls. To test this process, promising yeasts were cultivated in processed almond hulls, and quantity and quality of protein were determined.

## Materials and methods

### Compositional analysis of almond hulls

California and Nonpareil varieties of almond hulls harvested in 2018 were provided by the Almond Board of California. Air-dried hulls were coarse ground in a food processor and stored at room temperature. Hull samples used for compositional analyses were further Wiley milled to 40 mesh. Solids, ash, total water- and ethanol-soluble extractives, structural sugars, acid- and acid-insoluble lignin content were determined using published methods [21–23]. Extractives‐free hulls were generated by extractions of 40‐mesh milled dried hulls by refluxing in a Soxhlet apparatus for several days in water followed by 1 day in 95% ethanol. A separate series of extractions to determine soluble sugar content was conducted using 33% (w/w, d.b.) of 40 mesh milled almond hulls. Five sequential extractions were conducted; at each extraction step, almond hull slurries were incubated at 50°C in deionized water for one-hour with end-over-end rotation. Soluble sugars (sucrose, glucose and fructose) in the decanted extracts and solubilized structural sugars (glucose, xylose, arabinose, galactose and mannose) from acid hydrolysates were measured by high-performance liquid chromatography (HPLC). Milled and extractives-free hulls were analyzed for amino acid content.

### Production and characterization of almond hull hydrolysate

Almond hulls were liquefied by incubating 15% or 20% (w/w, d.b.) coarse-ground hulls in 0.1 M citrate buffer at pH 4.8 with 0.1 mg pectinase (Pectinex Ultra Clear; 9032-75-1 Novozymes) per gram of wet biomass at 50°C with end over end mixing for 3 days. The extent of liquefaction was assessed by measuring the free liquid volume after incubation. Solubilized sugars were measured by HPLC. Hydrolysates were frozen at -80°C then lyophilized for two days and weighed to determine soluble solids content and stored again at -80°C until amino acid analyses.

### Yeast screening

**Fig 1:**
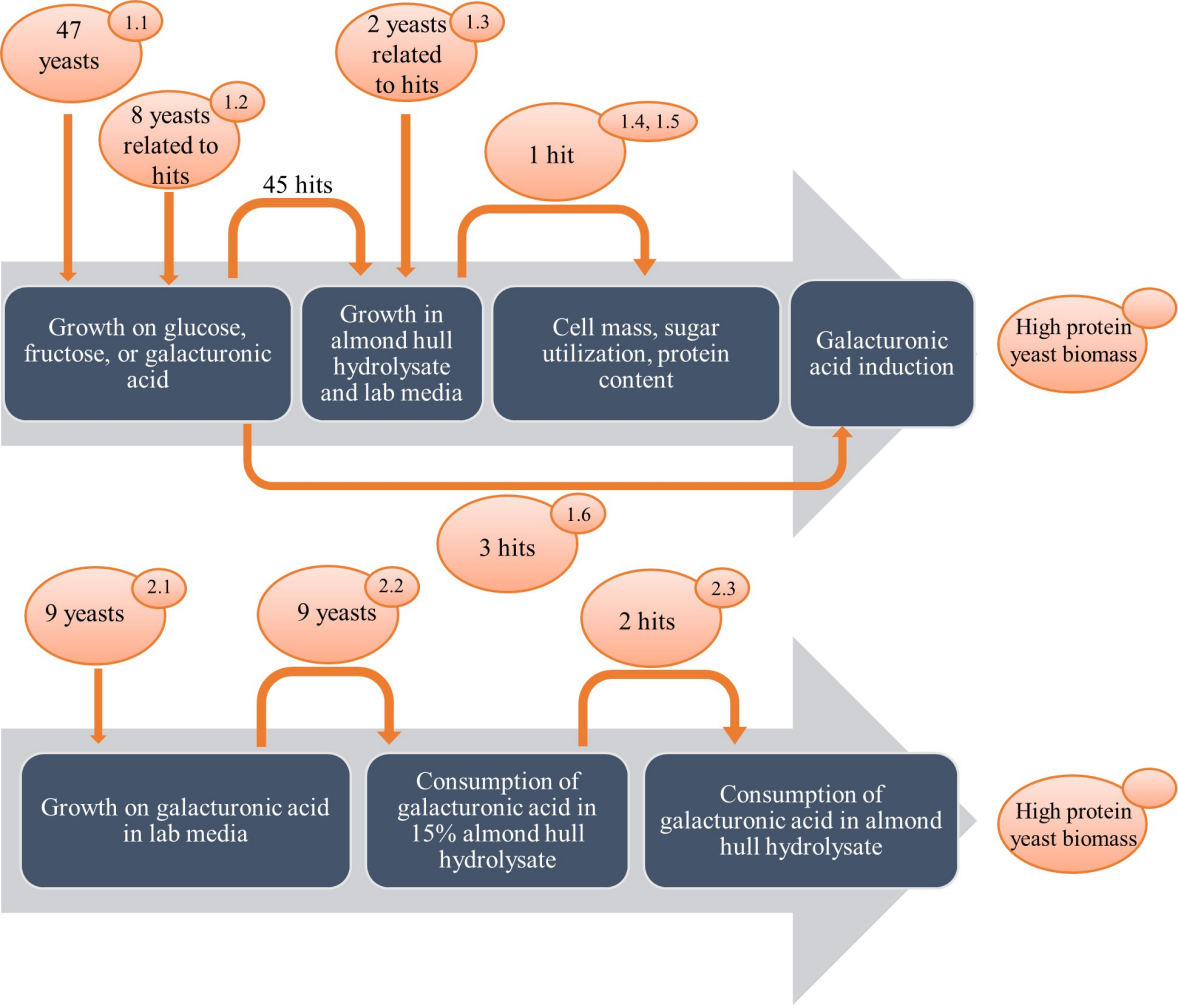
Screening strategy. A: Phase 1 and B: Phase 2.

All yeasts used in this study were acquired from the Phaff Yeast Culture Collection at the University of California Davis (collection identifier UCDFST, https://phaffcollection.ucdavis.edu). A total of 65 yeast strains (Table 1), including 47 strains from a previous study (Boundy-Mills et al., 2019), were used in a multi-phase screening process (Fig 1), described below [11]. The first phase involved 47 yeast strains and eight additional strains of genera *Zygoascus* and *Groenewaldozyma;* and the second part involved nine yeast strains. The screening strategy involved many steps to identify yeast species and strains able to consume carbon sources present in almond hull hydrolysate (AHH) (Fig 1).

**Table 1:** Yeast strains screened for growth in lab media and AHH (Phase 1).

Strain ID	Isolation source	Screening phase
*Barnettozyma californica* UCDFST 04–895	Adult female of *Bactrocera oleae*, Davis, California, USA	1.1, 1.3
*Barnettozyma californica* UCDFST 62–1022	Flux of *Magnolia* sp., California, USA	1.1, 1.3
*Candida boidinii* UCDFST 09–399	Banana fruit infested with *Drosophila melanogaster* larvae, Davis, California, USA	1.1, 1.3
*Candida boidinii* UCDFST 70–104	Olive brine, California, USA	1.1, 1.3
*Candida californica* UCDFST 04–933	Banana fruit infested with *Drosophila melanogaster* larvae, Davis, California, USA	1.1, 1.3
*Candida parapsilosis* UCDFST 80–184	Lowry’s teriyaki sauce, California, USA	1.1, 1.3
*Candida succiphila* UCDFST 12–108 = NRRL Y-11998 = ATCC 46049 = CBS 8003	Peach tree sap, *Amygdalus persica*, Yamanashi Prefecture, Japan	2.1, 2.2, 2.3
*Cyberlindnera jadinii* UCDFST 74–61 = DBVPG 7307 = ATCC 9950 = CBS 5609; NRRL Y-900	Fodder yeast, Unknown location	1.1, 1.3
*Debaryomyces hansenii* UCDFST 04–847	Adult female of *Bactrocera oleae*, California, USA	1.1, 1.3
*Debaryomyces hansenii* UCDFST 11–956	Pacifica raspberries infested with *Drosophila suzukii*, California, USA	1.1, 1.3
*Debaryomyces nepalensis* UCDFST 72–46 = CBS 5921 = NRRL Y-7534	Soil, Nepal	1.1, 1.3b
*Debaryomyces prosopidis* UCDFST 76–15 = DBVPG 7113	*Drosophila carbonaria* near flux of mesquite tree, Arizona, USA	1.1, 1.3
*Debaryomyces robertsiae* UCDFST 60–22 = CBS 2934 = NRRL Y-6670	Larva feed of *Xylocopa caffra*, South Africa	1.1, 1.3b
*Fibulobasidium inconspicuum* UCDFST 89–39	Unknown	2.1, 2.2
*Galactomyces candidus* UCDFST 09–568	*Drosophila* feed containing *Drosophila melanogaster* larvae, Davis, California, USA	1.1, 1.3
*Galactomyces candidus* UCDFST 52–260	*Drosophila persimilis*, California, USA	1.1, 1.3b
*Groenewaldozyma tartarivorans* UCDFST 12–178 = NRRL Y-27291 = CBS 7955	Dried lees of wine, Runa, Portugal	1.2, 1.6
*Groenewaldozyma tartarivorans* UCDFST 12–179 = NRRL Y-27500 = CBS 2985 = NRRL Y-11613	Peritoneal fluid of a dead guinea pig, Bogor, Indonesia	1.1, 1.2, 1.3
*Hasegawazyma lactosa* UCDFST 67–61 = ATCC 18177 = CBS 5827	Suburban atmosphere of Osaka, Japan.	2.1, 2.2
*Kazachstania lodderae* UCDFST 56–10	Soil, South Africa	1.1, 1.3
*Kluyveromyces lactis* UCDFST 69–8	*Quercus* sp. tree exudate, Japan	1.1, 1.3b
*Kluyveromyces lactis var*. *drosophilarum* UCDFST 81–535.1	Oak exudate, Arizona, USA	1.1, 1.3
*Kluyveromyces marxianus* UCDFST 05–822	Male walnut husk fly, California, USA	1.1, 1.3
*Meyerozyma caribbica* UCDFST 09–369	Banana fruit infested with *Drosophila melanogaster* larvae, Davis, California, USA	1.1, 1.3
*Meyerozyma caribbica* UCDFST 09–505	Banana fruit infested with *Drosophila melanogaster* larvae, Davis, California, USA	1.1, 1.3
*Meyerozyma caribbica* UCDFST 12–176 = NRRL Y-27274 = CBS 9966 = DBVPG 4519	Plant *Saccharum officinarum*, Cuba	1.1, 1.3
*Meyerozyma caribbica* UCDFST 44–4	Isolated from deteriorated Quartermaster items, Florida, USA	1.1, 1.3b
*Meyerozyma carpophila* UCDFST 04–829	Adult of *Bactrocera oleae*, California, USA	1.1, 1.3
*Meyerozyma carpophila* UCDFST 05–820	Male walnut husk fly, California, USA	1.1, 1.3
*Millerozyma aff*. *acaciae* UCDFST 09–254	Tunnel scrapings from oak tree, Petaluma California, USA	1.1, 1.3
*Myxozyma mucilagina* UCDFST 76–234.3 = ATCC 42171 = CBS 7070 = NRRL 11822	Rotting arm of agria cactus (*Stenocereus gummosus*), Todos Santos, Baja California Sur, Mexico	2.1, 2.2
*Myxozyma mucilagina* UCDFST 76–236.3 = ATCC 42174 = CBS 7071 = NRRL 11823 (T)	Rotting arm of agria cactus (*Stenocereus gummosus*), Todos Santos, Baja California Sur, Mexico	2.1, 2.2
*Ogataea naganishii* UCDFST 70–9 = ATCC 32418 = CBS 6429 = NRRL Y-7654 (T)	Exudate of *Camellia japonica*, Japan	2.1, 2.2, 2.3
*Pichia fermentans* UCDFST 67–546	Exudate of *Meliosma myriantha*, Tozawa, Japan	1.1, 1.3
*Pichia kluyveri* UCDFST 03–811	Banana fruit infested with *Drosophila melanogaster* larvae, Davis, California, USA	1.1, 1.3b
*Pichia kluyveri* UCDFST 04–151 = NRRL Y-48770	Banana fruit infested with *Drosophila melanogaster* larvae, Davis, California, USA	1.1, 1.3b
*Pichia kluyveri* UCDFST 09–390	Banana fruit infested with *Drosophila melanogaster* larvae, Davis, California, USA	1.1, 1.3
*Pichia manshurica* UCDFST 11–209	*Drosophila suzukii* larva from lab colony, Davis, California, USA	1.1, 1.3
*Pichia membranifaciens* UCDFST 68–102	Grape stem exudate, Davis, California, USA	1.1, 1.3
*Rhodotorula kratochvilovae* UCDFST 40–30	Unknown	2.1, 2.2
*Saccharomyces cerevisiae* UCDFST 09–448	Fermenting olive, Davis, California, USA	1.1, 1.2, 1.3
*Saccharomyces cerevisiae* UCDFST 40–148 = UCD VEN# 1148	Naturally fermenting vat of wine, Frei Bros. California, USA	1.1, 1.3
*Saccharomyces cerevisiae* UCDFST 75–4	A pineapple concentrate used in industry	1.1, 1.3
*Saccharomycopsis javanensis* UCDFST 55–49 = CBS 2555 = NRRL Y-1483 (T)	Soil, Java, Indonesia	2.1, 2.2
*Saturnispora diversa* UCDFST 10–266	*Drosophila suzukii* larva from an infested raspberry, Watsonville, California, USA	1.1, 1.3
*Scheffersomyces coipomoensis* UCDFST 92–97	Rotten material from *Alluritus moritana*, Hweihsi Forest, Taiwan	1.1, 1.3
*Scheffersomyces cryptocercus* UCDFST 73–1014	Bark beetle infestations of *Abies magnifica*, California, USA.	1.1, 1.3
*Schizosaccharomyces pombe* UCDFST 40–277 = ATCC 2476 = NRRL Y-164	Rum, Unknown location.	1.1, 1.3
*Slooffia tsugae* UCDFST 60–71 = ATCC 18802 = CBS 5038 = NRRL Y-5849	*Tsuga heterophylla* (western hemlock) bark frass, Siletz River, Oregon, USA	2.1, 2.2
*Suhomyces* aff. *xylopsoci* UCDFST 11–369	*Drosophila suzukii* adult from an infested raspberry, Watsonville, California, USA	1.1, 1.3b
*Wickerhamomyces anomalus* UCDFST 09–389	Banana fruit infested with *Drosophila melanogaster* larvae, Davis, California, USA	1.1, 1.3
*Wickerhamomyces anomalus* UCDFST 40–382	Dried pears, California, USA	1.1, 1.3
*Wickerhamomyces anomalus* UCDFST 82–2	Isolated from hot dog meat, Unknown location	1.1, 1.3
*Wickerhamomyces anomalus* UCDFST 83–21	Malt syrup	1.1, 1.3
*Zygoascus* aff. *bituminiphila* UCDFST 76–5	Flux of *Prosopis juliflora*	1.2
*Zygoascus* cf. *hellenicus* UCDFST 66–1031	Frozen grape fruit concentrate, California, USA	1.2
*Zygoascus* cf. *ofunaensis* UCDFST 91–573	Xylem of *Pinus ponderosa* tree, California, USA	1.2
*Zygoascus hellenicus* UCDFST 11–671 = Apr5Bb-3	Olive brine, Davis, California, USA	1.1, 1.2, 1.3b, 1.4, 1.5, 1.6
*Zygoascus hellenicus* UCDFST 70–40	Soil containing leaves and insects, Honduras	1.2
*Zygoascus hellenicus* UCDFST 83–734.3	*Opuntia stricta*, Bahamas, West Indies	1.2
*Zygoascus meyerae* UCDFST 11–728 = May5B-1	Pilot fermentation of Sicilian style olives, Davis, California	1.2, 1.6
*Zygoascus tannicolus* UCDFST 75–58 = ATCC 22263 = CBS 6067 = NRRL Y-7499	Isolated from tanning fluid in France	1.2
*Zygosaccharomyces baillii* UCDFST 70–117	Raw honey from comb, Davis, California, USA	1.1, 1.3
*Zygosaccharomyces rouxii* UCDFST 06–141	Dried prune energy bar, California, USA	1.1, 1.3

NRRL: USDA-Agricultural Research Service Culture Collection in Peoria, Illiois, USA; ATCC: American Type Culture Collection, Gaithersburg, Maryland, USA; CBS: Westerdijk Fungal Biodiversity Institute Collection, Utrecht, The Netherlands; DBVPG: University of Perugia, Italy; UCD VEN: UC Davis Viticulture and Enology Culture Collection. The taxonomic abbreviation “aff.” indicates that this yeast is likely a novel species that does not yet have a scientific name. The abbreviation “cf.” indicates that this yeast is either the indicated species or a close relative that does not yet have a scientific name.

Screening study: 1.1. Growth in 96 well plate with 250 μL broth containing glucose, fructose, or galacturonic acid; 1.2. Growth of genera *Zygoascus* and *Groenowaldozyma* on YNB agar with galacturonic acid: YNB + 0.5% glucose as a positive control, YNB + 2% galacturonic acid; YNB + 4% galacturonic acid, and YNB + a combination of 1.3% glucose, 0.7% fructose and 4% galacturonic acid, and 15% AHH agar; 1.3. Growth of 47 yeasts on 15% or 20% AHH in 2% agar plate with and without supplementation of nitrogen; 1.4. Analysis of amino acid composition and protein for *Zygoascus hellenicus* UCDFST 11–671 grown on AHH with/without nitrogen after 18 hours incubation; 1.5 Growing *Z*. *hellenicus* UCDFST 11–671 in AHH varied in nitrogen concentration, for cell dry mass estimation, sugar utilization and amino acid composition overtime; 1.6. Study to induce utilization of galacturonic acid; 2.1. Growing in YNB with galacturonic acid; 2.2. Growing in AHH with supplementation of nitrogen; 2.3. Confirmation of galacturonic acid utilization in AHH by *Candida succiphila* UCDFST 12–108 and *Ogataea naganishii* UCDFST 70–9.

The first phase was a six-step study to screen for yeasts that could grow well in AHH. Screening was carried out by measuring turbidity in liquid laboratory media or observation of growth on the surface of agar plates. A few yeasts were then grown in a larger volume of liquid media sufficient to measure carbohydrate consumption by HPLC.

The second phase involved screening for utilization of galacturonic acid in lab media and in AHH. Nine were selected that were reported to have ability to utilize galacturonic acid (Barnett, Table 1, Phase 2) [24]. These yeasts were grown in lab media with galacturonic acid as a sole carbon source and then in 15% AHH. Two strains were then selected for confirmation of galacturonic acid utilization in the AHH.

For all liquid culture screening phases in microplates, yeasts were revived from cryopreserved stocks onto PDA plates and incubated at room temperature for up to 3 days. Seed cultures were prepared in 250 μL of 0.67% yeast nitrogen base with 0.5% glucose in 96 well flat-bottom plates (Cat.# 3631, Costar®, New York, USA) sealed with breathable sterile sealing film (Cat. # BEM-1, Breathe-Easy, Diversified Biotech, Dedham, MA, USA) and incubated for one day at 30°C at 200 rpm in a VWR Microplate Shaker, 3 mm orbit. 5 μL of this seed culture was inoculated to 250 μL target media in a 96 well plate and incubated at 30°C at 200 rpm. Growth was determined by measuring the optical density at 600 nm (A600nm) in a microplate reader (VersaMax TM, Molecular Devices, LL, Sunnyvale California, USA) without prior shaking using two readings per time point for every 6 hours for the first two days then every 12 hours for the remaining observations.

### Indirect calculation of yeast cell biomass in turbid cultures

Growth of yeast in AHH could not be determined gravimetrically or by turbidity because AHH particulates. An indirect CFU to biomass conversion factor was therefore determined to correlate dry cell mass with colony forming units (CFU) per mL [24]. Yeast strain *Zygoascus hellenicus* UCDFST 11–671 was cultivated at 22°C in 0.67% (w/v) YNB, 0.5% w/v glucose. After 0, 6, 12, 18, 24, 36 and 48 hr of growth, 20 μL of serial diluted cultures (10^−1^ to 10^−6^ serial dilution) were plated on Rose Bengal Chloramphenicol Agar (RBCA, Cat. #DF1831-17-4, BD Difco™, Fisher Scientific, Fair Lawn, NJ, USA) plate and CFU were counted daily. Cell dry weight was determined by centrifuging the whole culture at 3,220 x g for 10 min, washing twice with sterile water, freezing at –80°C, and then freeze drying at −46°C, 0.133 mbar (Freezone® 4.5 L Freeze Dry System Model 7750020, Labconco®, Kansas City, MO, USA) until constant mass was achieved. The ratio of cell dry mass to CFU/mL was calculated and used to estimate the yeast mass grown in AHH.

### Part 1. Screening of a total of 55 yeast strains for growth and utilization of sugar, and resulting amino acid composition

#### Phase 1.1: Screening of yeasts for ability to consume glucose, fructose, and galacturonic acid

Forty-seven yeast strains (Table 1, Phase 1.1) belonging to potentially high protein species were tested in duplicate in 96-well flat bottom plates for ability grow in 0.67% yeast nitrogen base (YNB, Cat #239,210, Difco™, Becton, Dickinson and Company, Sparks, MD, USA) containing 0.5% (w/v) D-glucose (Cat. #D16–3, Fisher Scientific, Fair Lawn, NJ, USA), D(-)-fructose (Cat. #47740, Fluka BioChemika, Buchs, Switzerland), D(+)-galacturonic acid (Cat.# 324–50342, Wako Chemicals USA, Inc., Richmond, VA, USA), or no sugar as a negative control [11].

#### Phase 1.2: Ability of Zygoascus and Groenowaldozyma to consume galacturonic acid on agar plates

Two yeast strains in genera *Zygoascus* and *Groenewaldozyma* that performed well in Phase 1.1 plus eight more yeast strains of these genera (Table 1, Phase 1.2) were tested for ability to grow on agar plates containing various combinations of carbon sources present in almond hulls. Of various combinations tested, plates containing 15% AHH and 2% agar had the best combination of hydrolysate handling and smooth surface texture. Additional plates included YNB with 0.5% glucose; YNB with 2% galacturonic acid; YNB with 4% galacturonic acid; and YNB with 1.3% glucose, 0.7% fructose and 4% galacturonic acid to mimic the main sugar composition in AHH (synthetic almond hull hydrolysate or SynAHH). Growth was visually scored on a scale from 0 (no growth) to 6 (strongest growth) based on the overall dimension of the colony including diameter, margin and elevation.

#### Phase 1.3: Screening yeast for ability to consume AHH on agar plates

Forty-seven yeast strains (Table 1, Phase 1.3.) were tested for growth on 15% or 20% AHH in 2% agar, without and with supplementation of nitrogen (0.5% w/v ammonium sulfate, Cat. #10325410, Fisher Scientific, Fair Lawn, NJ, USA). Growth was visually scored on a scale of 0 (no growth) to 5 (strongest growth) based on the colony diameter. Growth of nine strains (Table 1, Phase 1.3b) was confirmed by the same method with greater distance between yeasts.

#### Phase 1.4: Analysis of protein quantity and quality of yeast grown in liquid or solidified AHH without and with added nitrogen

Protein content of yeast grown on the surface of AHH agar was determined. One loopful of *Zygoascus hellenicus* UCDFST 11–671 grown on a PDA plate at 30°C for 1 day was suspended in 5 mL sterile water, for a seed culture with OD600 nm of 1.75. 50 μL of seed culture was spread on the surface of 20% AHH solidified with 2% agar and incubated at 30°C for 18 hours. Cells were then scraped off the surface of the agar plate, freeze dried and weighed.

Protein content of yeast grown in liquid media was also determined. As a positive control, 500 μL of the seed culture described above was added to 9.5 mL potato dextrose broth (PDB) in a 15 mL Bio-reaction tube and incubated at 30°C shaking at 200 rpm for 24 hours in. Cells were centrifuged, washed twice with sterile DI water to remove the residual nutrients and amino content of the yeast cell pellet was determined. For the test conditions, 0.5 mL of the same seed culture from each yeast tested was added to two different media, i.e. 9.5 mL of 10% AHH with or without ammonium sulfate in a 15 mL Bio-reaction tube and incubated at 30°C shaking 200 rpm for 24 hours before freeze-drying the whole culture.

The amino acid content of AHH, AHH liquid media with and without yeast, and nitrogen-supplemented AHH with and without yeast was analyzed at the UC Davis Proteomics Core facility following the method described below.

#### Phase 1.5: Cell dry mass, sugar utilization and amino acid composition of Zygoascus hellenicus UCDFST 11–671 grown in lab media and AHH

The same yeast was cultivated at 22°C in 15% liquid AHH supplemented with 0, 0.25, 5, or 10 g/L ammonium sulfate (Cat.# A702-500, Fisher Scientific, Fair Lawn, NJ, USA), and CFU/mL determined at the same time points. The calculated ratio of cell dry mass to CFU/mL described above section (Indirect calculation of yeast cell biomass in turbid cultures) was multiplied by CFU/mL of yeast in hydrolysate to estimate cell dry mass of yeast grown in AHH. Amino acid content was analyzed at the UC Davis Proteomics Core facility.

#### Phase 1.6: Effort to induce utilization of galacturonic acid

To test whether cultivation of the seed culture in galacturonic acid induces utilization of galacturonic acid, three yeast strains that grew well in galacturonic acid in laboratory media, were pre-grown with and without galacturonic acid. One loopful each of three yeast strains (Table 1, Phase 1.6) grown overnight on PDA were suspended in 5mL saline Tween® 80 (Cat. # P1754-500ML, Sigma-Aldrich, Burlington, MA, USA) diluent in 5mL tubes (Cat. # 0030119460, Eppendorf™, Fisher Scientific, Fair Lawn, NJ, US).250 μL of the seed cultures were inoculated to two 4.75mL YNB media pH 5.5 with different carbon sources, i.e. 2.5% glucose or 2.5% galacturonic acid pH 5.5 in 18 mm culture tubes (Cat. # 14-961-32, Fisher Scientific, Fair Lawn, NJ, USA). These six seed cultures were incubated for one day in a rotary drum at 22°C, then 250 μL were added in triplicate to 4.75 mL each of three different media in 15 mL Bio-Reaction tubes (Cat. # 229471, Celltreat, Shirley, MA, USA): YNB media with 2.5% glucose pH 5.5, YNB media with 2.5% galacturonic acid pH 5.5, or YNB media with 2.5% glucose and 2.5% galacturonic acid pH 5.5. Cultures were incubated at 30°C for three days at 200 rpm. One of the triplicate cultures of each condition was processed at 1, 2 and 3 days by centrifuging at 3,220 x g for 10 min, sterile filtering the supernatant with 0.45 um membrane (Cat.# SLHU033RB, Millex®-HV, Tullagreen, Co.Cork, IRL). The residual sugars in the supernatants were analyzed with HPLC.

### Phase 2. Galacturonic acid consumption and protein content

#### Phase 2.1: Consumption of galacturonic acid in lab media

Nine yeasts (Table 1, Phase 2.1) were selected for this phase, based on known ability of the species to consume galacturonic acid [24–26]. After reviving the yeast from cryopreserved stocks on PDA plates, grown 3 days at 30°C, one loopful of yeast cells was suspended in 3 mL sterile DI water. 150 μL of this suspension was inoculated into 3 mL of 0.67% (w/v) YNB media with 2.5% (w/v) galacturonic acid in an 18 mm culture tube and incubated in a rotary drum at 22°C for four days. Growth was visually scored daily on a scale of 0 (no growth) to 4 (most turbid growth) and floculation was noted, as previously described [24]. Galacturonic acid in the spent supernatant was quantified by HPLC [22,23].

#### Phase 2.2: Growth and consumption of galacturonic acid in AHH

The nine yeast strains (Table 1, Phase 2.2) were also grown in 15% AHH by preculturing in 0.67% (w/v) YNB with 0.5% galacturonic acid for two days at 22°C. 500 μL were added to 9.5 mL of 15% AHH, pH 5.5 in 15 mL bioreaction tubes, and incubated 72 hours at 22°C shaking at 200 rpm. The CFU/mL was determined at 0, 24, 48 and 72 hr by plating serial dilutions on PDA square plates and incubating at 22°C. Residual sugars in the spent supernatant were identified and quantified by HPLC [22,23].

#### Phase 2.3: Confirmation of galacturonic acid utilization in AHH

One loopful of yeast cells of strains *Candida succiphila* UCDFST 12–108 and *Ogataea naganishii* UCDFST 70–9 cultivated up to 3 days at 22°C on PDA were suspended in 3 mL sterile 0.85% NaCl, 0.01% Tween 80 diluent in a sterile 5 mL tube (Cat. # 0030119460, Eppendorf™, Fisher Scientific, Fair Lawn, NJ, USA). One hundred and fifty μL of the inoculum was added to 3 mL YNB with 2.5% galacturonic acid in 15 mL Bio-Reaction tubes and incubated at 30°C for one day. This seed culture was centrifuged at 3,220 x g for 5 min, and the cells were concentrated to 1 mL by removing the 2 mL supernatant from the tube. OD600nm ranged from 2.6–3.5. 0.5 mL of the seed culture was added in triplicate to 9.5 mL 15% AHH with 0.5% ammonium sulfate, pH 5.5 in 50 mL Bio-Reaction tubes (Cat. #229475, Celltreat, Shirley, MA, USA), and incubated at 30°C for three days. CFU and reducing sugar analysis were analyzed as described.

### Carbon and nitrogen content analyses

Wiley milled raw almond hulls and hydrolysate were analyzed for total carbon and nitrogen content at the UC Davis Analytical Laboratory by the combustion method. Samples were oxidized by flash combustion and the resultant gases analyzed [27]. Dried material was combusted in an induction furnace coupled with a thermal conductivity detector and infrared detector (TruSpec CN Analyzers). This method has a nitrogen detection limit of 0.2 g kg^−1^ and carbon detection limit of 1 g kg ^−1^.

### Amino acid and protein content analysis

Amino acid contents for raw and extractives-free almond hulls, hydrolysate, and yeast cultures were analyzed at the UC Davis Proteomics Core facility. Approximately 10 mg dry samples were used per analysis. Briefly, each sample was hydrolyzed for 24 h *in vacuo* in 6 N HCl at 110°C and then vacuum‐dried. The hydrolysate/culture was suspended in a precise quantity of diluent (NorLeu as internal standard) and then subjected to strong ion exchange with sodium citrate buffer mobile phase to separate the resulting amino acids, which were detected by absorption in the visible spectrum using a Hitachi L‐8800 amino acid analyzer. Response factors were calculated from standards and each amino acid was identified and quantified. In addition, the standard used for calibration is validated against NIST standard ref. material 2389a in each sequence.

### Hydrolysate analysis by HPLC

Analysis of soluble components for composition analysis, hydrolysate characterization and post-fermentation evaluation was performed using a Shimadzu HPLC with a refractive index detector and photodiode array detector [23]. All samples were adjusted to pH 4–7, filtered using 0.45 μm syringe filters before analysis and were ran in triplicate. Monosaccharides and sucrose were separated using a Biorad Aminex HPX-87P column (Cat. #1250098) at 80°C using nanopure water at 0.6 mL/min.

## Results

### California and Nonpareil almond hulls are carbon rich but nitrogen poor feedstock

Hulls from two almond varieties, California and Nonpareil, were analyzed for their composition (Table 1). Both varieties contained similar amounts of ash, extractives, protein, structural carbohydrates (glucan, xylan, galactan, arabinan, mannan and galacturonan), and soluble carbohydrates (sucrose, glucose and fructose). Consistent with previously reported compositions [28–30], the hulls are rich in carbohydrates and deficient in protein, and therefore have high carbon to nitrogen ratios (C:N = 49 and 50 for California, and Nonpareil, respectively). The higher galacturonan, arabinan, and acetyl content indicate significant pectin content in the hulls. The total protein content of California and Nonpareil hulls, 3.2% and 3.0% (d.b.) respectively, were similar (Table 2). Essential amino acids comprised 31% of the total protein of California hulls, and 28% of Nonpareil hulls (Table 3). In both almond hull varieties, the protein consisted largely of insoluble structural proteins and thus was inaccessible without disruption and degradation of the cell wall fractions (see Supplemental Information).

**Table 2:** Composition of California and Nonpareil almond hull samples.

	Almond Hull Variety	
	California	Nonpareil
Ash	53 (0)	58 (0)
Total water extractives	312	336
*Sucrose* ^*1*^	*9 (0)*	*1 (0)*
*Glucose* ^*1*^	*120 (3)*	*89 (1)*
*Fructose* ^*1*^	*114 (0)*	*75 (0)*
Ethanol extractives	47	42
Acid-insoluble lignin	143 (35)	231 (7)
Glucan	172 (36)	145 (1)
Xylan	54 (11)	32 (3)
Galactan	59 (11)	28 (1)
Arabinan	42 (8)	8 (2)
Mannan	7 (1)	5 (3)
Galacturonan	136 (33)	179 (40)
Acetyl	4 (3)	15 (14)
Protein^2^	32	30
Total	1030 (63)	1079

All values are reported as g/kg dry basis with standard deviations in parentheses from triplicate analyses. ^1^ Sucrose, glucose and fructose contents were determined from a cold-extraction process to minimize degradation loss. The abundance of glucose and fructose relative to sucrose is likely due to hydrolysis during extraction. Differences between the soluble sugar content and total water extractives likely reflect incomplete sugar extraction. ^*2*^Protein content was determined from the sum of amino acids (Table 3).

**Table 3:** Amino acid composition of California and Nonpareil almond hulls.

Amino Acid	California (g/kg)	Nonpareil (g/kg)
Asparagine/Aspartic Acid (Asx)	10.5	13.9
Threonine (Thr)*	1.1	0.9
Serine (Ser)	1.4	1.2
Glutamine/Glutamic Acid (Glx)	4.0	2.1
Proline (Pro)	2.3	2.3
Glycine (Gly)	1.3	0.9
Alanine (Ala)	1.3	1.3
Valine (Val)*	1.3	1.4
Isoleucine (Ile)*	0.9	0.6
Leucine (Leu)*	1.7	1.1
Tyrosine (Tyr)	0.8	0.6
Phenylalanine (Phe)*	1.1	0.7
Histidine (His)*	0.7	0.5
Lysine (Lys)*	1.2	1.0
Arginine (Arg)	1.5	0.8
Cysteine (Cys)	0.4	0.3
Methionine Sulfone (Met)*	0	0
Tryptophan (Trp)*	0.4	0.3
Total Protein	31.7	29.9
Total Essential Amino Acids	10.2	7.6
% of Protein as Essential	32%	26%

All values are given g/kg of almond hulls. Essential amino acids are denoted with asterisks.

### Enzymatic liquefaction of almond hulls to produce carbon-rich hydrolysate

Conventionally, soluble sugars from plant biomass are obtained by water-extraction, a water and energy intensive process [29–34]. This study employed the strategy of enzymatically hydrolyzing the pectin fraction of almond hulls, the hygroscopic polysaccharide responsible for high water holding capacity of plant cell walls, to solubilize sugars while minimizing water usage (Table 4). California hulls demonstrated greater post-liquefaction volume and concentration of glucose and fructose release from hydrolysates. The optimal conditions for reducing input costs, improving soluble glucose and fructose release, and maintaining mechanically favorable consistency for use with fermentation setups were 15% w/w, dry basis almond hull solids in pH 5, 0.1 M citrate buffer with 0.1 mg of pectinase/g almond hull reacted for 3 days in a well-mixed, 50°C setup.

**Table 4:** Composition of AHH with the optimal solids loading, enzyme loading, and reaction time for solubilization of glucose.

Amino Acid	Concentration (g/L)
Cellobiose	1.2
Glucose	23.1
Xylose^1^	<0.1
Galactose	0.8
Arabinose	5.0
Mannose^1^	<0.1
Fructose	17.9
Galacturonic Acid	51.2
Lactic Acid	0.3
Formic Acid	29.5
Acetic Acid	0.7
Levulinic Acid	55.4
Hydroxymethylfurfural	0.6
Furfural^1^	<0.1

^1^ below detectable limits.

### Phase 1. Screening of yeasts for utilization of AHH

#### Growth of yeast in lab media (YNB with glucose, fructose, or galacturonic acid)

**Fig 2:**
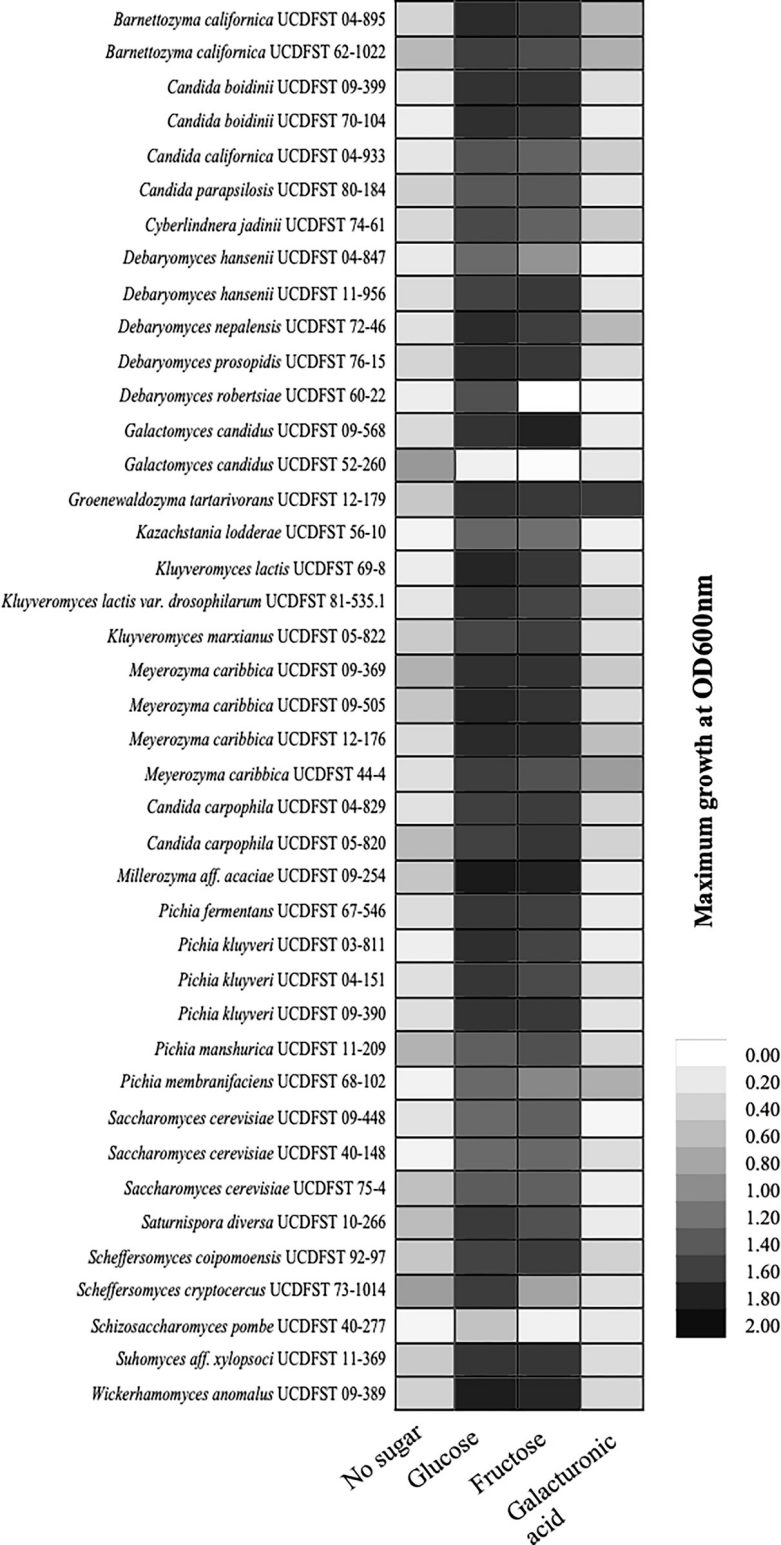
Screening Phase 1.1 results. Maximum growth as reflected as OD600nm by each yeast tested in 250 uL YNB media with glucose, fructose or galacturonic acid.

As expected, most yeasts could grow well in glucose or fructose as carbon sources. However, only two out of 47 yeast strains tested in Phase 1.1, *Zygoascus hellenicus* UCDFST 11–671 and *Groenowaldozyma tartarivorans* UCDFST 12–179, could grow well in lab media containing galacturonic acid as the sole carbon source (S1 File; Fig 2). The two yeast strains were isolated from olive brine and guinea pig, respectively. To see if this trait is specific to these two genera, in Phase 1.2 an additional eight strains belonging to these two genera were then cultivated at two different temperatures (Table 1, Phase 1.2, Fig 2, S1 File).

**Fig 3:**
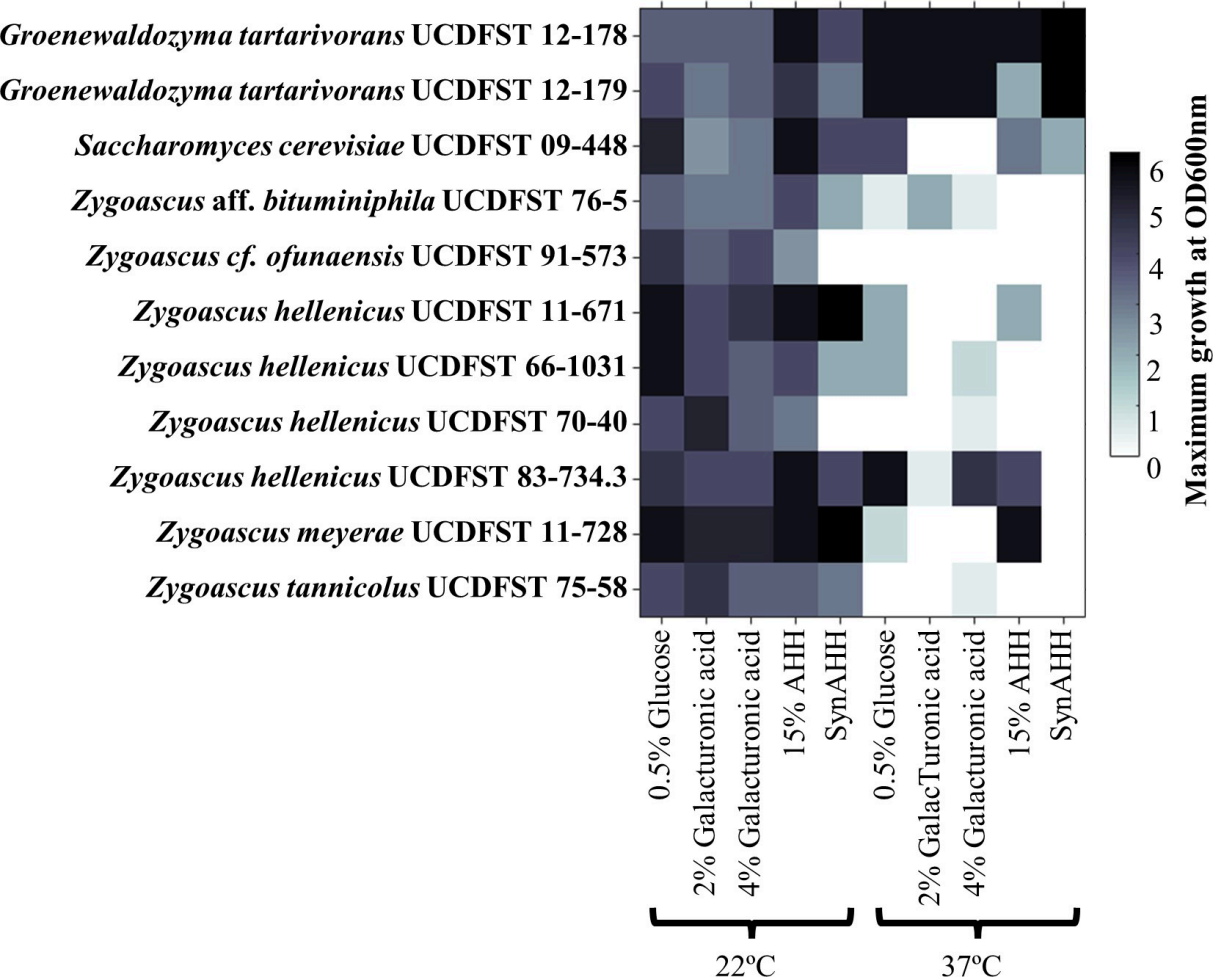
Screening Phase 1.2 results. Growth of *Zygoascus* and *Groenowaldozyma* strains at two different temperatures (22°C and 37°C) and different carbon sources (0.5% glucose, 2 and 4% galacturonic acid, and 15% AHH and SynAHH.

Most yeasts tested in Phase 1.2 grew to higher density in most media at 22°C than at 37°C, except strains *G*. *tartarivorans* UCDFST 12–178 and 12–179 (Fig 3). *Zygoascus hellenicus* UCDFST 11–671 had the highest density growth in media with fructose although most strains were able to utilize fructose (Fig 2). Two yeast strains, *Z*. cf. *ofunaensis* UCDFST 91–573 and *Z*. *hellenicus* UCDFST 70–40, did not grow in SynAHH. *Zygoascus hellenicus* UCDFST 11–671 was therefore selected for further analysis.

### Phase 1.3: Growth of yeast on AHH with and without nitrogen supplementation

**Fig 4:**
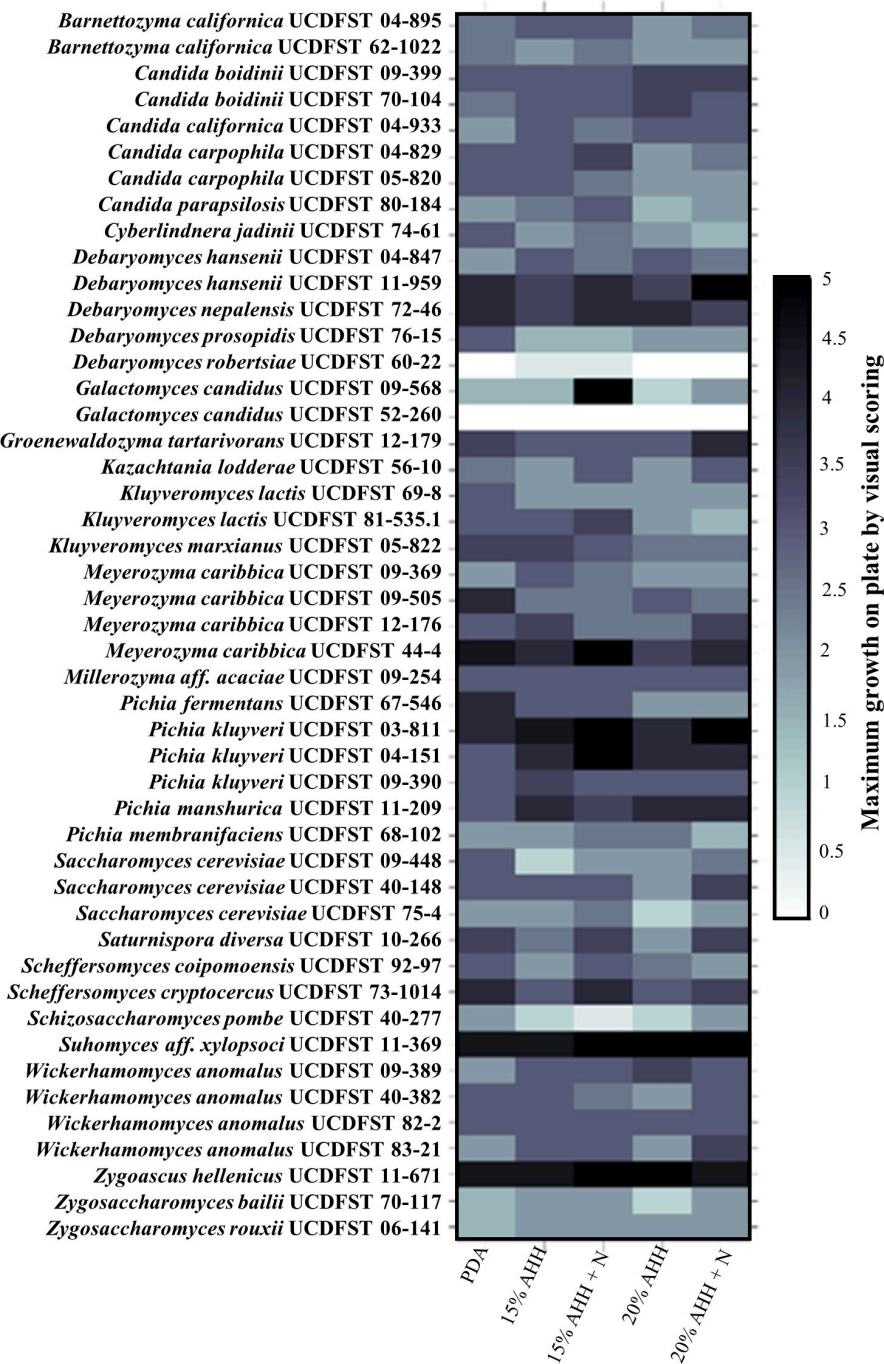
Phase 1.3 results. Relative growth of 47 yeasts on 15% or 20% (v/v) AHH agar plates, with or without 0.5% supplemented nitrogen in the form of 0.5% (w/v) ammonium sulfate (“+N”), scored visually from 0 (no growth) to 5 (most dense growth). Media, left to right: PDA (positive control), 15% AHH, 15% AHH + nitrogen, 20% AHH, 20% AHH + nitrogen.

Growth of 47 yeasts was compared on lab media (PDA) and AHH agar with varying concentrations of hydrolysate and nitrogen supplementation (Fig 1, Table 1, Phase 1.3, S2 File, and Fig 4). *Zygoascus hellenicus* UCDFST 11–671 and *S*. aff. *xylopsoci* UCDFST 11–369 grew well in all media. Most yeasts grew better in AHH with nitrogen supplementation. *Pichia kluyveri* strains UCDFST 03–811, 04–151, 09–390 and *P*. *manshurica* UCDFST 11–209 grew better in AHH than in PDA, the positive control media. Because crowding could affect yeast growth, robust growth of the nine most promising candidates was confirmed by plating further apart. Yeast strain *Z*. *hellenicus* UCDFST 11–671 was selected for further analysis.

### Phase 1.4: Amino acid content of *Zygoascus hellenicus* UCDFST 11–671

**Fig 5:**
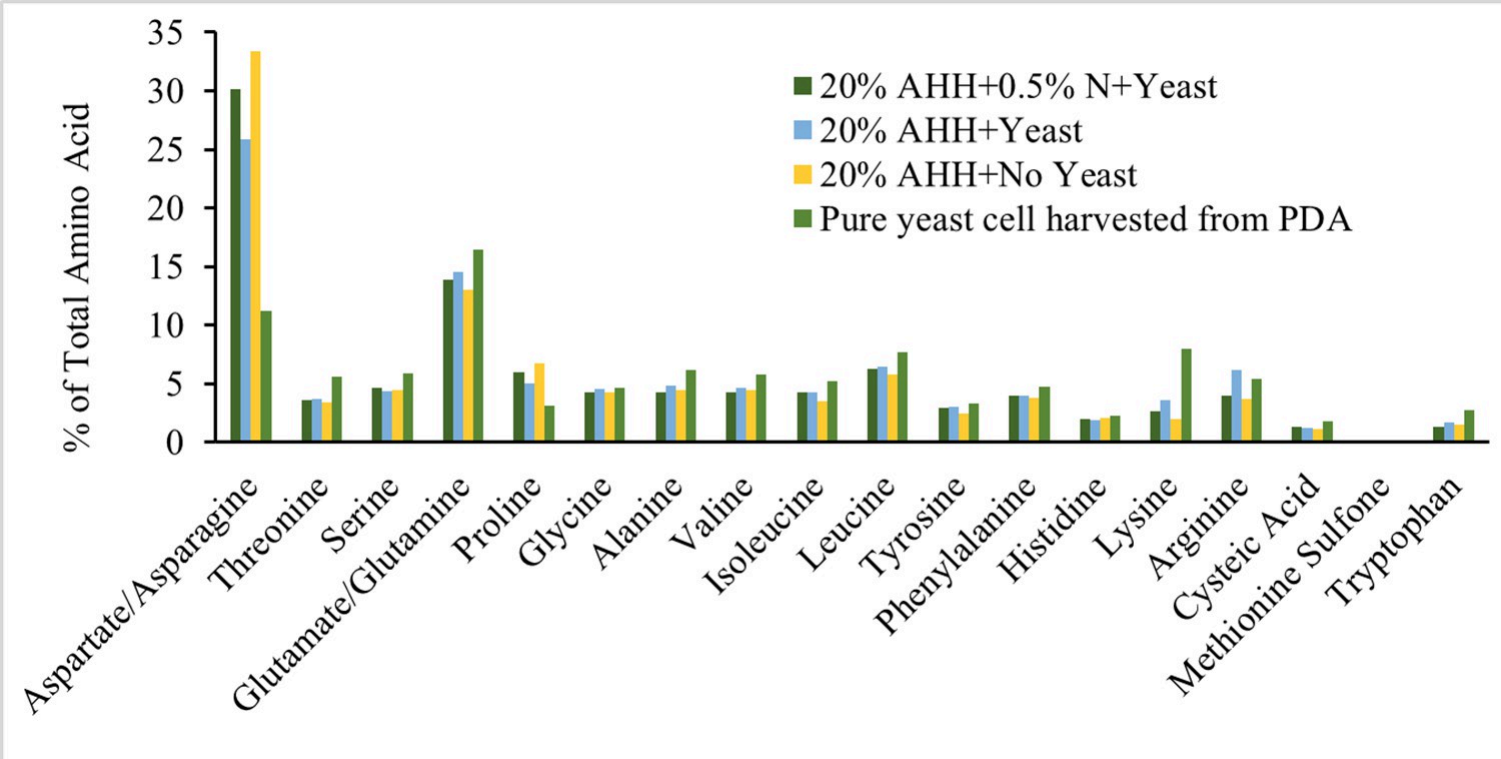
Percent of total amino acids of AHH media with and without yeast inoculation (No yeast) and of yeast cell biomass harvested off the surface of AHH agar with or without ammonium sulfate, and from the surface of rich media PDA.

*Zygoascus hellenicus* UCDFST 11–671 produced more visible yeast biomass on PDA than on 20% AHH agar without added nitrogen (S3 File). Compared to the AHH media without yeast, yeast cell biomass harvested off the surface of the AHH and PDA agar plates had higher essential amino acids lysine, tryptophan and valine, and lower levels of less desired amino acids including aspartate and asparagine. Yeast cell biomass harvested from the surface of AHH agar was contaminated with particulates from agar, due to the rough surface of the agar, so yeast biomass analysis likely includes some contaminating agar. Yeasts biomass grown on PDA had higher levels of most amino acids than other treatments, except for aspartate/asparagine and proline. The overall protein content was highest in yeast biomass grown in agar media with 20% AHH without addition of supplemental nitrogen with 3.91% w/w, 1.39% w/w higher than that of the media with no yeast. Adding 0.5% w/w supplemental nitrogen in the form of ammonium sulfate resulted in 3.02% w/w protein for the media containing 20% AHH and inoculated with yeast and 2.58% w/w protein for the media without yeast inoculation (Fig 5).

**Fig 6:**
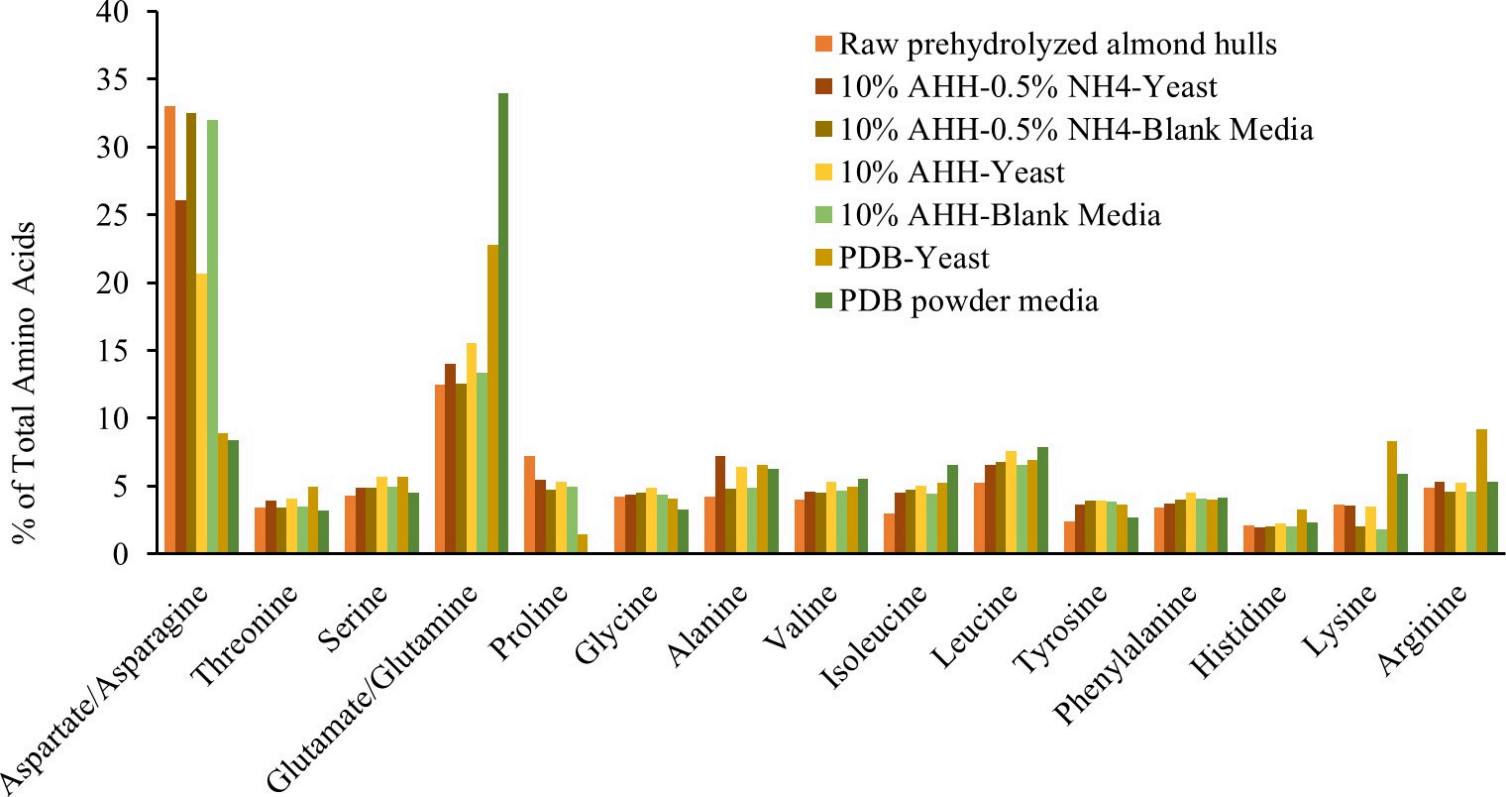
Amino acid composition of *Zygoascus hellenicus* UDCFST 11–671 grown in liquid 10% (w/v) almond hull hydrolysate (AHH), 10% AHH with 0.5% ammonium sulfate (AHHN), or potato dextrose broth (PDB) for one day at 200rpm, 30C. Media containing only PDB or AHH with and without added nitrogen included to demonstrate change in amino acid profile.

Experiments with agar media presented difficulties in evaluating yeast cell biomass due to the rough texture of the agar. To allow determination of CFU/mL at 10^6^ of yeast biomass, a similar experiment was performed using liquid media with lower solids loading to facilitate pipetting. Cultures were grown in 10% AHH with and without added nitrogen, and in potato dextrose broth (PDB) as a control. The control culture, the 10% AHH culture, and the 10% AHH culture with 0.5% ammonium sulfate had 38.5, 44, and 51 x 10^6^ CFU/mL, respectively. For total protein percent on a dry weight basis, the 10% AHH with no yeast had 2.26%, with yeast had 2.31%, and with 0.5% added ammonium sulfate and yeast had 2.68%. The expanded amino acid profiles for the 10% AHH with and without yeast and added nitrogen are shown below in Fig 6.

CFU/mL and total protein were highest in AHH with added nitrogen and yeast. Relative to the media without yeast, the yeast cultures were higher in most amino acids including essential amino acids: threonine, serine, glutamine/glutamate, alanine, valine, isoleucine, leucine, leucine, lysine, and arginine. Non-essential, abundant amino acids like aspartate/asparagine were lowered in cultures with yeast. Cysteine, methionine and tryptophan were not measured in this phase.

### Phase 1.5: Optimization of cell mass, protein content, amino acid composition and sugar utilization

**Fig 7:**
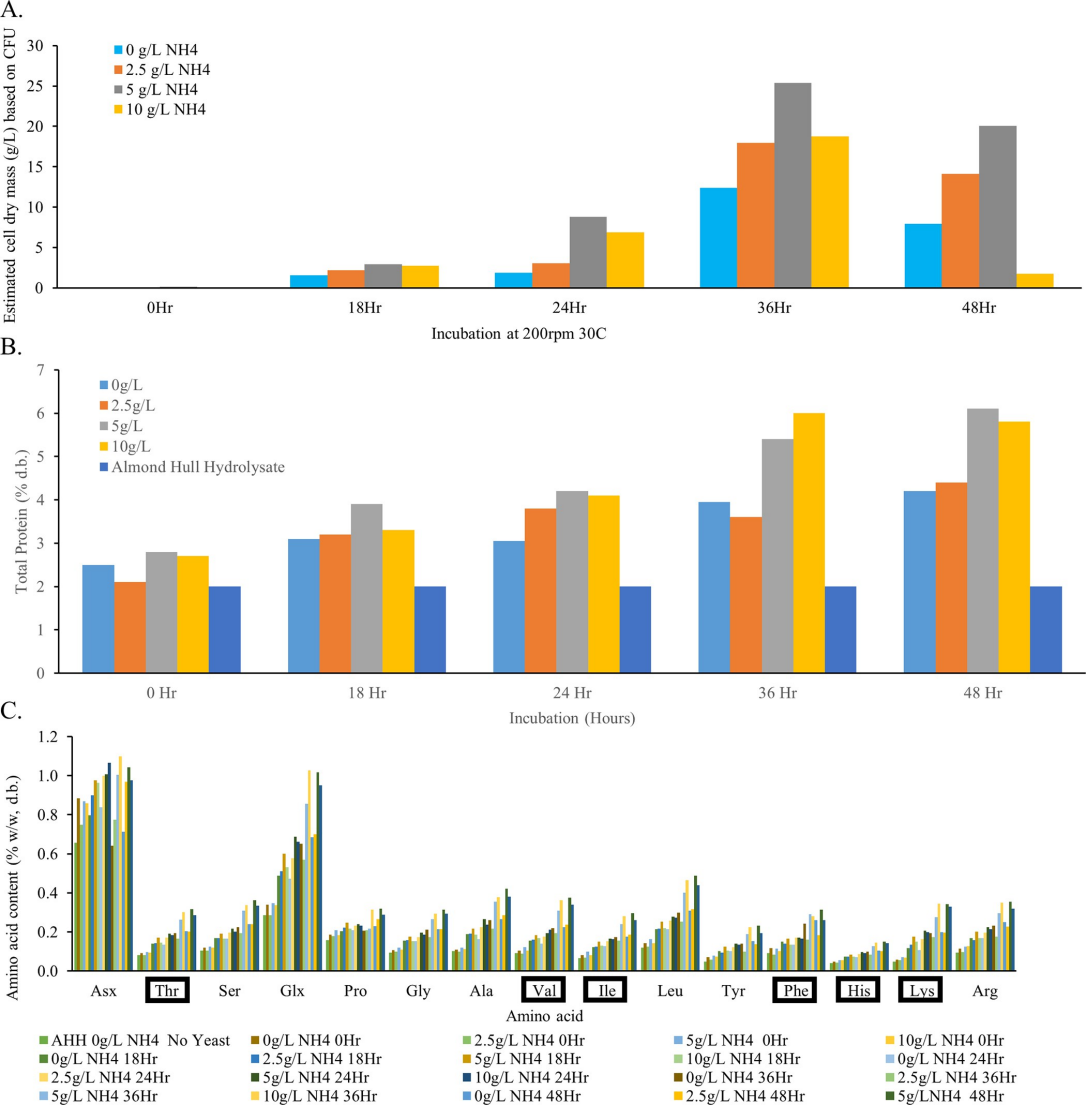
Cell mass, protein content, amino acid composition, and sugar utilization of *Zygoascus hellenicus* UCDFST 11–671 grown in 15% AHH with and without supplemented ammonium sulfate; A: Dry cell mass calculated from CFU/mL measurements (CFU data is presented in S4 File); B: % (w/w) total protein of AHH yeast cultures calculated by summing dry mass of 18 individual amino acids; C: Amino acid composition (omitting cysteine and methionine) with essential amino acids indicated in boxes; D: Concentrations of major monosaccharides over culturing time containing 5 g/L added ammonium sulfate.

Based on the indirect CFU to biomass calculation, 1.2 x 10^9^ CFU/mL was equivalent to 25 g/L dry cell mass (S4 File). This conversion factor was used to approximate dry cell mass in the yeast/AHH slurries. Fig 7 depicts the effects of the amount of supplemented ammonium and incubation time on *Z*. *hellenicus* UCDFST 11–671 growth (as CFU/mL), sugar utilization, and protein accumulation. Calculated total cell mass was highest with 5 g/L ammonium sulfate and 36 hours incubation (Fig 7A). Total protein (Fig 7B), calculated by adding the concentrations of the 18 amino acids measured in this experiment (Fig 7C), was also highest under this growth condition. The concentrations of all amino acids, including essential amino acids, increased over time (Fig 7C). Total protein increased from 2.1% in AHH to 6.3% (w/w) with 5 g/L ammonium sulfate at 48hr in this study, a 3-fold increase. This study confirmed that addition of ammonium and cultivation of yeast increased the total protein content of AHH.

The most abundant sugars in AHH were glucose (26 g/L), fructose (25 g/L), and galacturonic acid (30 g/L). The concentrations of these sugars plus xylose and sucrose over time are shown in Fig 7D. *Zygoascus hellenicus* UCDFST 11–671 reduced glucose and fructose to low levels within 18 and 24 hours respectively, but the galacturonic acid concentration was unchanged at 48 hours, indicating this yeast was unable to consume galacturonic acid.

### Phase 1.6. Induction of galacturonic acid utilization by selected yeasts

**Fig 8:**
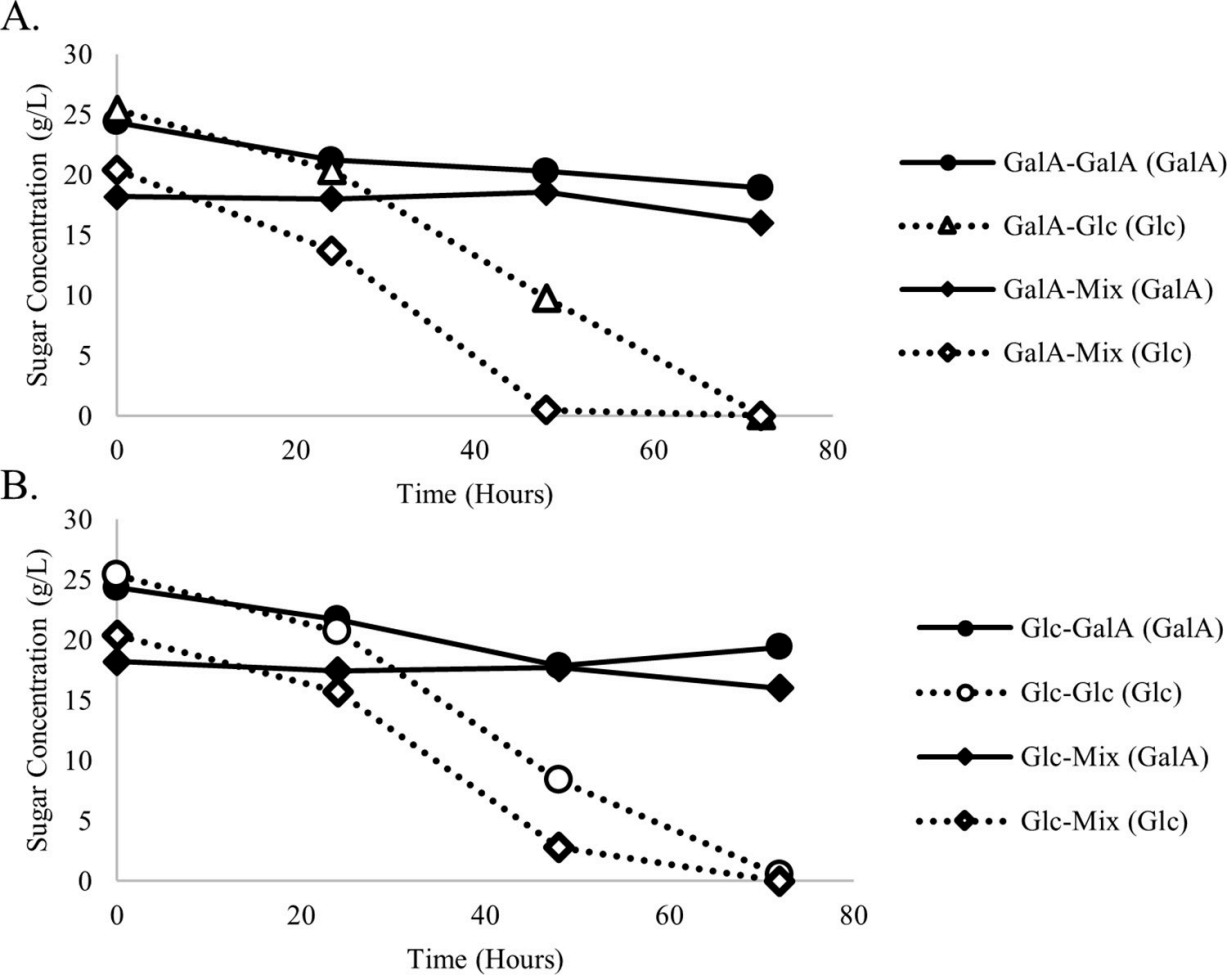
Sugar consumption of Zygoascus hellenicus UCDFST 11–671 pre-grown in A: Galacturonic acid (GalA) and B: Glucose (Glc) before transferring to media containing glucose, galacturonic acid, or a mixture as the sole carbon sources.

To test whether consumption of galacturonic acid could be induced, three yeasts were pre-cultured in galacturonic acid before inoculating into medium containing both galacturonic acid and glucose. In addition to *Z*. *hellenicus* UCDFST 11–671, two additional yeasts, *Z*. *meyerae* UCDFST 11–728 and *G*. *tartarivorans* UCDFST 12–178, that were able to consume galacturonic acid (Fig 2) were added for this screening step. When seed cultures were cultivated in lab media containing galacturonic acid or glucose, all three yeasts grew better in glucose than galacturonic acid (Fig 8). In the subsequent test media containing galacturonic acid, glucose, or both, *G*. *tartarivorans* UCDFST 12–178 grew better than the other two yeast strains both in the absence and presence of galacturonic acid, however all three strains demonstrated growth regardless of glucose or galacturonic acid pre-culture. Sugar analysis results indicated that growing yeasts in galacturonic acid prior to culturing in the target media in an attempt to induce utilization of galacturonic did not efficiently improve the ability of these yeasts in using the target sugar. The yeast utilized glucose more efficiently than other sugars when glucose is present (Fig 8B and 8C).

### Phase 2. Selection of more yeast strains to test for ability to utilize galacturonic acid

#### Phase 2.1

**Fig 9:**
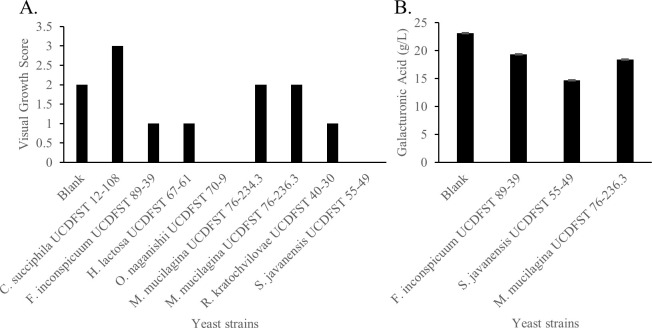
A: Visual score of turbidity of nine yeast strains, grown for 72 hr at 22C in YNB + 2.5% galacturonic acid; and B: Galacturonic acid remaining in spent media after growth for 4 days at 22C in YNB + 2.5% galacturonic acid.

The strains of *Zygoascus* and *Groenowaldozyma* tested in Phase 1 utilized glucose and fructose but not galacturonic acid in either liquid lab media or in AHH. In Phase 2, nine additional yeast strains (Table 1, Phase 2.1, 2.2 and 2.3) were selected based on the published ability of these yeast species to assimilate galacturonic acid [23]. When grown in 3 mL of YNB with galacturonic acid (Fig 9), *Fibulobasidium inconspicuum* UCDFST 89–39 showed growth after one day of incubation, followed by five strains (*Candida succiphila* UCDFST 12–108, *Rhodotorula kratochvilovae* UCDFST 40–30, *Myxozyma mucilagina* UCDFST 76–236.3, *Hasegawazyma lactosa* UCDFST 67–61, *Ogataea naganishii* UCDFST 70–9) after two days, and another strain (*Saccharomycopsis javanensis* UCDFST 55–49) after three days (Fig 9A). Strain *S*. *cerevisiae* UCDFST 55–49 had flocculant growth. One of the *Myxozyma mucilagina* strains, UCDFST 76–234.3, did not show any growth in this media. Three strains of interest, chosen based on their growth, were able to utilize galacturonic acid (Fig 9B): *Fibulobasidium inconspicuum* UCDFST 89–39 and *Myxozyma mucilagina* UCDFST 76–236.3 started to show growth density one day after inoculation; whereas *Saccharomycopsis javanensis* UCDFST 55–49 had flocculation.

**Fig 10:**
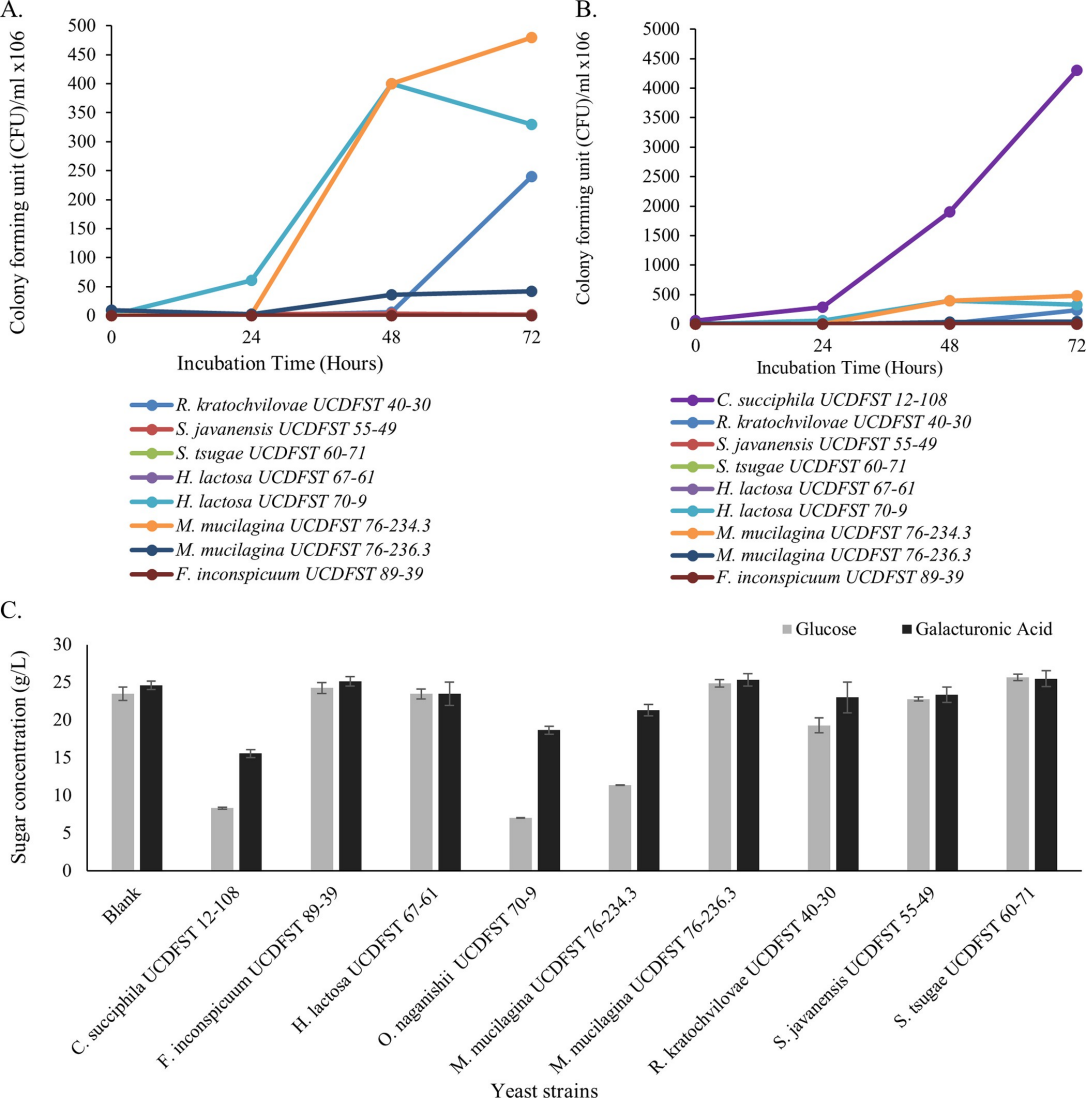
Growth and utilization of glucose and galacturonic acid by nine yeast strains in 15% AHH supplemented with 1% ammonium sulfate. Growth depicted as colony forming unit (CFU)/mL for A: All strains and B: All strains except *Candida succiphila* UCDFST 12–108 to show growth of other strains more clearly; C: Remaining glucose and galacturonic acid in the spent media.

The AHH contained particulates made it impossible to directly weigh yeast cell mass yield. Therefore, a plate count method was used to evaluate yeast biomass indirectly. Strain *Candida succiphila* UCDFST 12–108 had by far the strongest growth (Fig 10A and 10B). This strain also showed the highest utilization of both glucose and galacturonic acid when grown in the almond hulls (Fig 10C). Both data suggested that yeasts that had growth by CFU, utilized sugar in the AHH.

**Fig 11:**
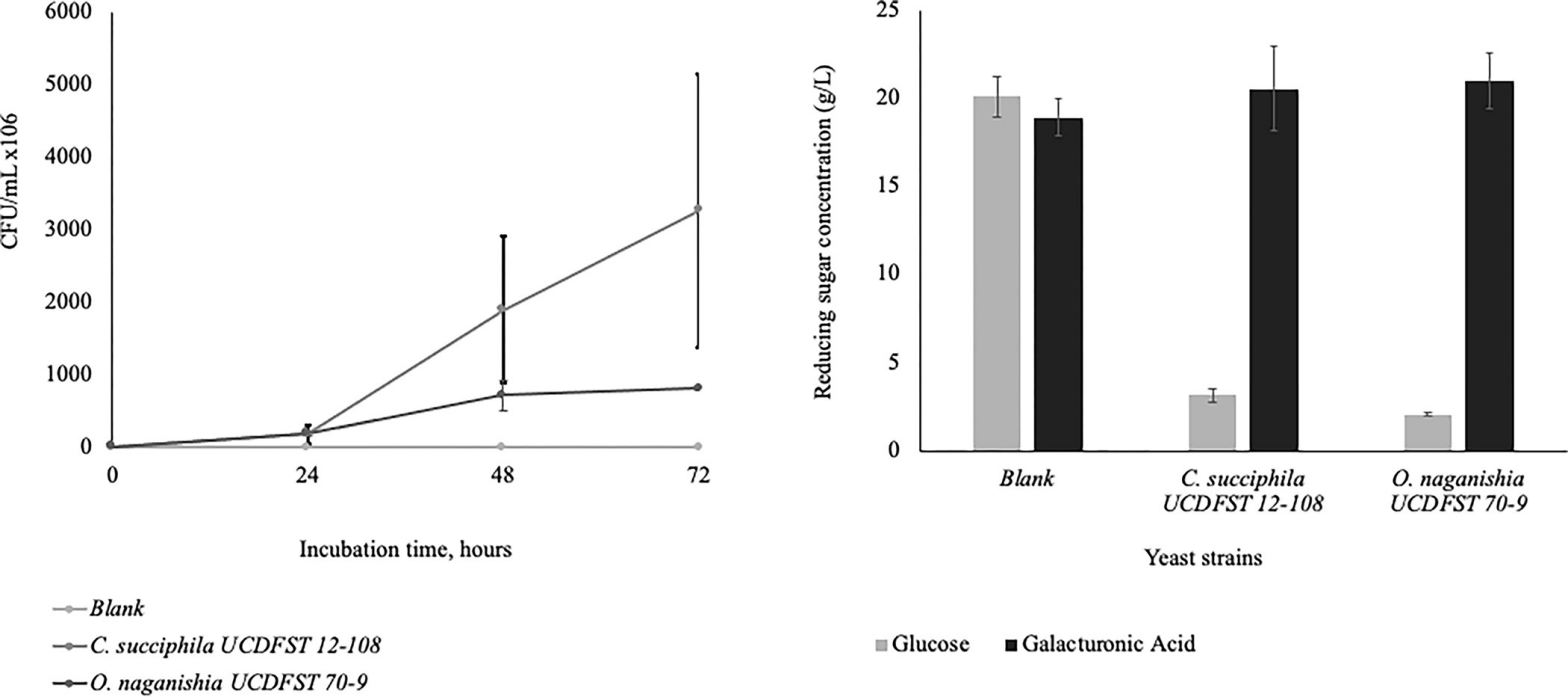
Growth and utilization of sugars in the AHH by two yeast strains *Candida succiphila* UCDFST 12–108 and Ogataea *naganishii* UCDFST 70–9. A: Growth of the yeast depicted as CFU/mL and B: Concentrations of glucose and galacturonic acid remaining in the hydrolysate after growth.

To determine whether preculturing these yeasts in galacturonic acid as the sole carbon source could induce subsequent utilization of galacturonic acid in AHH, seed cultures were cultivated in lab media containing galacturonic acid then inoculated into AHH. Initial results indicated that some galacturonic acid induction may occur in only two of the strains examined. Further examination confirmed that galacturonic acid was consumed in the seed culture and the yeasts were able to grow and consume glucose in AHH, however the galacturonic acid was not consumed within 3 days of growth (Fig 11). Further work is needed to determine whether galacturonic acid would be consumed after longer incubation.

## Discussion

### Current alternatives for protein in animal feed

Guidelines for composition of animal feed for various livestock are published and updated regularly by the US Department of Agriculture NCRS. For example, beef cattle feed should contain between 6% and 9.3% dry weight of crude protein, depending on the age of the animal [35], however, the quality of the protein is just as important as the quantity. Total crude protein is an inferior indicator of overall cattle feed quality. Essential amino acids, especially lysine, cysteine and methionine, have the greatest influence on cattle growth [36,37]. Protein ingredients in livestock and aquaculture include soybean meal, which is 50 to 58% crude protein, 3% lysine, and 1.3% cysteine plus methionine by weight [38]; fish meal, which is 68% crude protein; and grain distillers dried yeast which is 52% crude protein and 2.3% lysine by dry weight [39]. The current wholesale feedstuff price of soybean meal is $328–342 per ton.

Competitive microbial proteins must maximize protein quality while minimizing production costs including transportation. Both the feedstock generation site, and the livestock production site, should be physically near the yeast conversion facility. Almond hulls are a strong candidate for bioconversion because they are an abundant wastestream from almond production in California, which is also a major producer of dairy cattle, aquaculture and poultry. The current market wholesale price of almond hulls is $290–320 per US Ton [40]. Valorization of almond hulls into a protein-enriched feed stock for dairy cattle for precision feeding with specialized amino acid profiles would greatly improve the wholesale price of almond hulls as specific essential amino acid content increases.

An optimized yeast conversion process must improve the protein content and quality to approach or surpass that of existing protein content and quality should be comparable or superior to other feed ingredients, while keeping the total costs comparable. Conversion of most or all the carbon sources in the feedstock would improve the economic balance.

### Composition of California and Nonpareil almond hulls

Both varieties of almond hulls, California and Nonpareil, contained low amounts of protein (2–3% d.b.) but abundant carbohydrates, which are desirable for downstream yeast fermentation. Low nitrogen content (<1% d.b.) dictates a need for nitrogen supplementation prior to yeast fermentation.

### Production and composition of AHH

Enzymatic liquefaction of both almond hull varieties, California and Nonpareil, was achieved using pectinase and/or cellulase with varying incubation times ranging from one to three days, enzyme loadings and almond hull loadings. Hydrolysates produced from California hulls had higher glucose and fructose concentrations than Nonpareil hulls under the same liquefaction conditions. Increased incubation time coupled with milder temperatures for enzymatic deconstruction of structural polysaccharides resulted in higher volumes of hydrolysate without compromised sugar contents. Low pectinase enzyme loadings (0.1 mg enzyme/g almond hull) produced similar sugar compositions to high enzyme loadings (5 mg enzyme/g almond hull) regardless of incubation time, however galacturonic acid concentration and hydrolysate volume increased with longer liquefaction reactions. Although the sugar content of hydrolysate did not change, increasing liquid release while maintaining the same concentration equates to freeing more sugar into the hydrolysate, increasing accessibility of feed for downstream yeast. Using pectinase resulted in a significant increase in liquefaction volume over use of only cellulase while resulting in similar liquid release and hydrolysate composition to samples produced using a combination of pectinase with cellulase. Thus, pectinase-only loadings were chosen for the process to minimize costs, while relatively long incubation times of 36- and 48-hour reactions.

Increasing the solids loading of almond hulls for liquefaction resulted in increased hydrolysate sugar content but created mass transfer complications in downstream yeast processes related to distribution and measurement of yeast within AHH culture volumes.

### Yeast screening strategy

As demonstrated in our previous screening studies, a yeast screening schema is often guided and modified based on discoveries made during the study [41–44]. Two key results that guided the screening strategy in this study were that the AHH contained significant quantities of galacturonic acid, and that few yeasts in the initial screening sets could consume this compound.

Three main sugar components in the AHH were glucose, galacturonic acid, and fructose. Two yeasts strains used in this study were able to utilize glucose, fructose and galacturonic acid individually in lab media. However, when they were cultivated in AHH, glucose and fructose were consumed completely but galacturonic acid was not. Efforts to induce utilization of galacturonic acid by *Z*. *hellenicus* UCDFST 11–671 in the presence of glucose and fructose by cultivating the seed culture in galacturonic acid as the sole carbon source did not improve the utilization of galacturonic acid in the mixed substrate. Three other yeast strains could only partially utilize the galacturonic acid in lab media. This observation is consistent with prior publications. Growth inhibition by galacturonic acid has been reported in *S*. *cerevisiae* grown in hydrolysate originally rich in pectin source, such as sugar beet pulp and orange peel [45]. Protzko et al. (2018) suggested that α-(1,4)-linked d-galacturonic acid (d-galUA) utilization by *S*. *cerevisiae* was inhibited by the presence of high glucose concentration and low pH in the rich D-galUA hydrolysate [46]. The yeast was then genetically engineered to be able to co-utilize the D-galUA and glucose. Future studies could include screening wild yeasts for a high-flux transporter to allow uptake of d-galUA in low and high pH hydrolysate, and in the presence of high glucose.

In this proof-of-concept trial, the calculated ratio of grams of yeast cell biomass produced to grams of sugar consumed (the yield coefficient) was approximately 0.56. This compares favorably to optimized industrial processes, which are in the range of 0.45 to 0.55 [47]. If additional input carbon (galacturonic acid) were consumed, the ratio may be unchanged but the overall yield could be increased.

### Effect of nitrogen supplementation on total protein content and amino acid composition

Because almond hulls are low in nitrogen, supplementation with added nitrogen was necessary to produce acceptable protein yields. The strain *Zygoascus hellenicus* UCDFST 11–671 was selected to test the effect of varying levels of nitrogen supplementation and incubation times on protein content. It is generally accepted that yeasts that are actively dividing contain the highest protein content, because they have a high number of ribosomes. In this study, the total protein content by dry weight of the material was increased approximately three-fold from 2% in unprocessed almond hulls to over 6% in the yeast/AHH slurry. While the protein content of the yeast could not be measured directly because it was not possible to separate yeast cells from the AHH, the protein content was approximated to be about 24%, based on the calculated yeast cell mass yield of 25 g/L. Examples of literature values for protein content in various species of yeasts grown under optimized conditions for animal feed include 45.6% for brewer’s yeast *Saccharomyces cerevisiae*, 54.3% for *Cyberlindnera jadinii* (formerly called *Candida utilis*, or torula yeast), and 50.5% for *Yarrowia lipolytica* [48]. Grain distiller’s dried yeast is comprised of 52% crude protein [39]. Based on these values, improvement of the process developed here could result in further increase in protein content.

Two strategies could further improve protein yield: greater consumption of galacturonic acid, and higher yeast protein content, through different yeast species or strains, different pre-culturing conditions, or both. The additional yeast species able to consume galacturonic acid that were identified in this study could be the subject of future studies. If any of these yeasts have ability to completely consume galacturonic acid, the yeast cell mass yield could theoretically increase from 25 to 40 g/L.

Only one nitrogen supplement, ammonium sulfate, was used in this exploratory study. Other nitrogen sources such as nitrate, nitrite, or nitrogen-rich waste streams have not yet been examined. If both the galacturonic acid consumption were complete, yielding a calculated 40 g/L yeast cell biomass, and the protein content in the yeast cells increased from 24% to 40%, the total protein yield would be 16 g/L.

### Advantages of yeast as a high-protein feed ingredient

In addition to quantity of protein, the quality of protein is also a key factor in animal feed. Soybean meal is approximately 3% lysine by dry weight [38], and grain distiller’s dried yeast is roughly 2.3% lysine by dry weight [39]. While the lysine content of the AHH increased from less than 0.1 to over 0.3% (w/w) after yeast growth, the total lysine concentration is still well below that of major protein sources for animal feed. Further improvements are needed.

Observed benefits of incorporating brewer’s yeast (*Saccharomyces* cerevisiae) in feed for cattle, hogs or poultry include increased milk yields, faster piglet weight gain, improved feed conversion ratio in poultry, and increased egg production and quality [49–54]. While three species of yeasts are currently used as a protein ingredient in livestock and poultry feed, a fourth species, *Phaffia rhodozyma*, has been used as a source of the pigment astaxanthin for salmon in aquaculture. There are over 2,000 yeast species that have not yet been explored for applications in animal nutrition. Comparison of the composition of a large number of non-GMO yeast strains is possible due to preservation of a broad range of species in culture collections such as the Phaff Yeast Culture Collection. Optimizing total protein content and quality as well as co-products such as antimicrobial compounds, vitamins or sterols may improve the economic viability of this technology.

## Conclusions

Almond hulls are a low value, high sugar agricultural byproduct that could potentially be an appropriate feedstock for cultivating yeast for animal feed or other applications. A large proportion of the carbon in almond hulls is in the form of pectin, which has three forms, the most common of which is a polymer of galacturonic acid. These results indicate that few yeast species are able to efficiently utilize galacturonic acid. Further work is needed to develop an economically viable conversion process, which may involve mixed or sequential cultures.

## Supporting information

S1 File Growth of yeast in 250uL YNB media with glucose, fructose or galacturonic acid. (PDF)

S2 File Growth of 47 yeasts on 2% agar. A: Potato dextrose as positive control; B: 15% almond hull hydrolysate agar; C: 20% almond hull hydrolysate agar; D: 15% almond hull hydrolysate agar with 0.5g/L ammonium sulfate; E: 20% almond hull hydrolysate agar with 0.5g/L ammonium sulfate; F: Map of yeast on the plate and the strain ID is presented in heatmap Fig 4.
(PDF)

S3 File A. Texture of 20% almond hull hydrolysate. Growth at 18 hours after inoculation of Zygoascus hellenicus UCDFST 11–671 on B. PDA; C. 20% AHH agar; and D. 20% AHH agar with 0.5% ammonium sulfate.
(PDF)

S4 File Colony forming unit of Zygoascus hellenicus UCDFST 11–671 grown in 15% almond hull hydrolysate varied in nitrogen concentration at different timepoints of incubation was used to estimate the cell mass. (PDF)
