# Supplementary figures and images for: Production of high protein yeast using enzymatically liquefied almond hulls
Source: PLoS One. 2023 Nov 15;18(11):e0293085. doi: 10.1371/journal.pone.0293085 (PMC10651018; doi:10.1371/journal.pone.0293085)

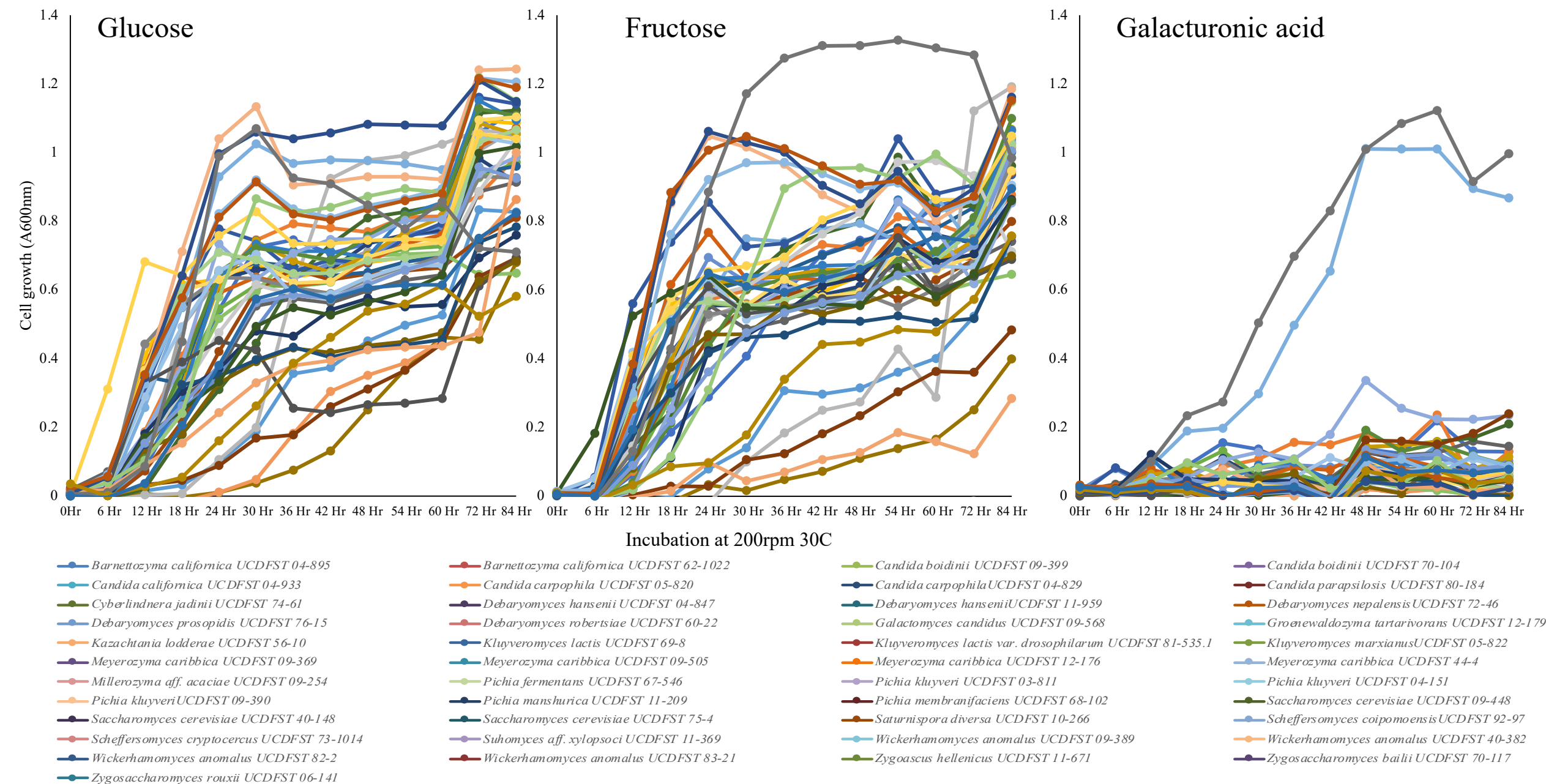

S1 File. Growth of yeast in 250uL YNB media with glucose, fructose or galacturonic acid.

Supplement: S1 File — (PDF) [file pone.0293085.s001.pdf]
