# Supplementary material for: Production of high protein yeast using enzymatically liquefied almond hulls
Source: PLoS One. 2023 Nov 15;18(11):e0293085. doi: 10.1371/journal.pone.0293085 (PMC10651018; doi:10.1371/journal.pone.0293085)
Supplement: S2 File — A: Potato dextrose as positive control; B: 15% almond hull hydrolysate agar; C: 20% almond hull hydrolysate agar; D: 15% almond hull hydrolysate agar with 0.5g/L ammonium sulfate; E: 20% almond hull hydrolysate agar with 0.5g/L ammonium sulfate; F: Map of yeast on the plate and the strain ID is presented in heatmap Fig 4. (PDF) [file pone.0293085.s002.pdf]

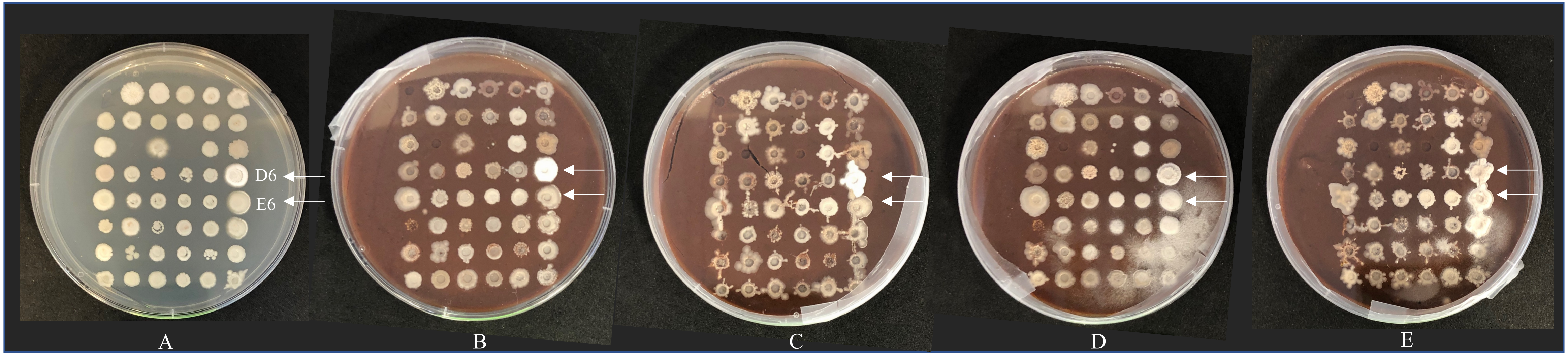

D6: *Z. hellenicus* UCDFST 11-671 E6: *S. aff. xylopsoci* UCDFST 11-369

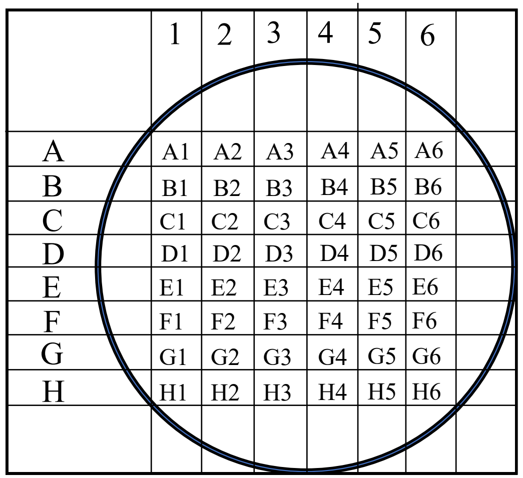

F

S2 File. Growth of 47 yeasts on 2% agar. A: Potato dextrose as positive control; B: 15% almond hull hydrolysate agar; C: 20% almond hull hydrolysate agar; D: 15% almond hull hydrolysate agar with 0.5g/L ammonium sulfate; E: 20% almond hull hydrolysate agar with 0.5g/L ammonium sulfate; F: Map of yeast on the plate and the strain ID is presented in heatmap Fig 4.
