# Supplementary material for: Production of high protein yeast using enzymatically liquefied almond hulls
Source: PLoS One. 2023 Nov 15;18(11):e0293085. doi: 10.1371/journal.pone.0293085 (PMC10651018; doi:10.1371/journal.pone.0293085)
Supplement: S3 File — Growth at 18 hours after inoculation of Zygoascus hellenicus UCDFST 11–671 on B. PDA; C. 20% AHH agar; and D. 20% AHH agar with 0.5% ammonium sulfate. (PDF) [file pone.0293085.s003.pdf]

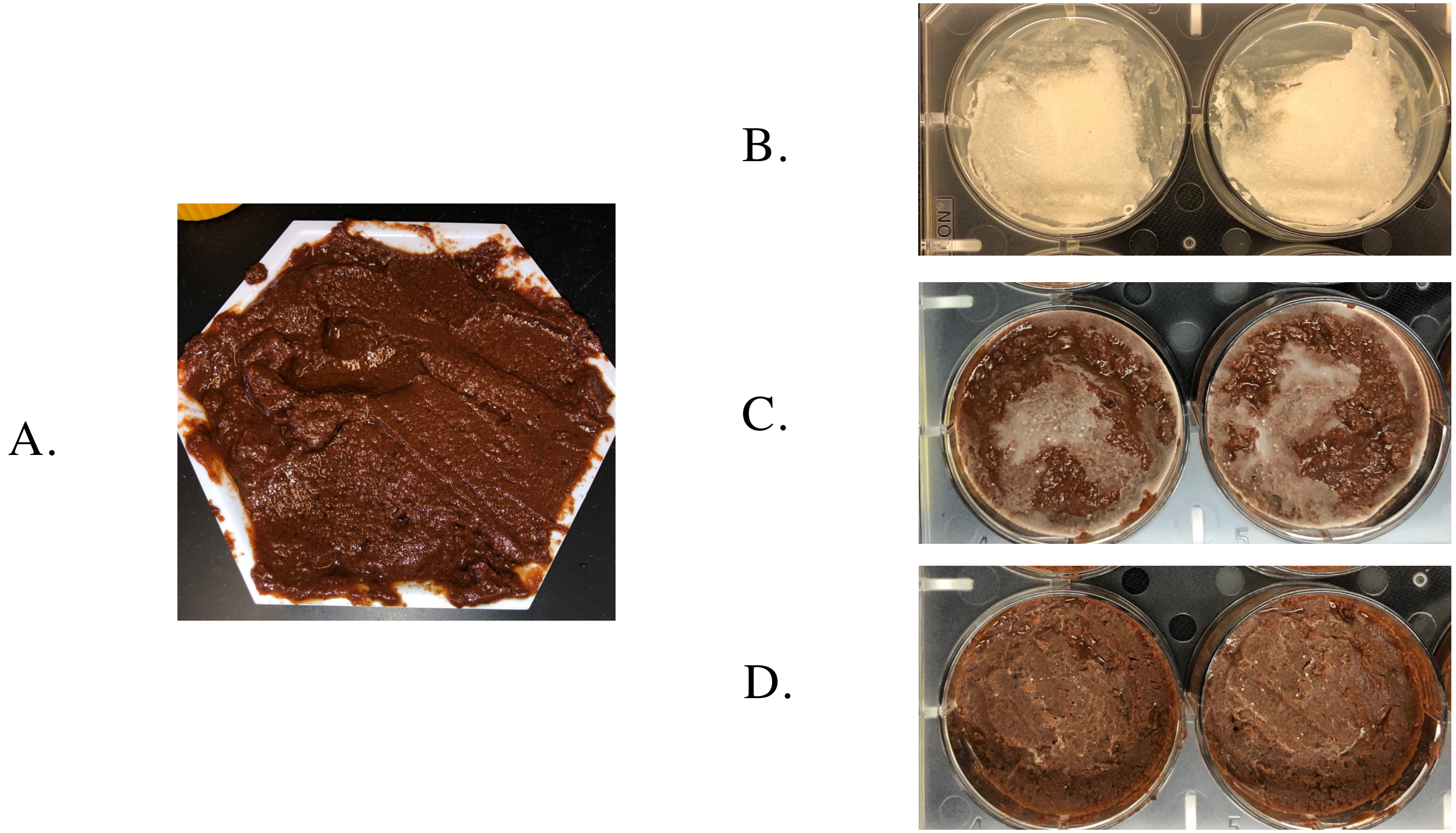


S3 File. A. Texture of 20% almond hull hydrolysate. Growth at 18 hours after inoculation of Zygoascus hellenicus UCDFST 11-671 on B. PDA; C. 20% AHH agar; and D. 20% AHH agar with 0.5% ammonium sulfate.
