# Supplementary material for: Production of high protein yeast using enzymatically liquefied almond hulls
Source: PLoS One. 2023 Nov 15;18(11):e0293085. doi: 10.1371/journal.pone.0293085 (PMC10651018; doi:10.1371/journal.pone.0293085)
Supplement: S4 File — (PDF) [file pone.0293085.s004.pdf]

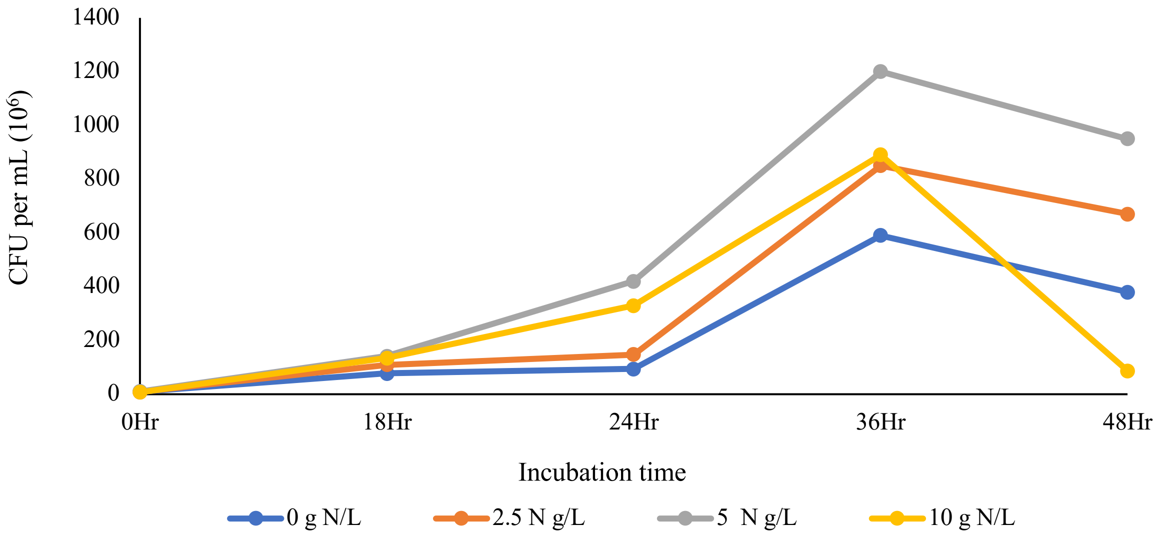


S4 File. Colony forming unit of *Zygoascus hellenicus* UCDFST 11-671 grown in 15% almond hull hydrolysate varied in nitrogen concentration at different timepoints of incubation was used to estimate the cell mass
